# *Mycobacterium tuberculosis* bloodstream infection prevalence, diagnosis, and mortality risk in seriously ill adults with HIV: a systematic review and meta-analysis of individual patient data

**DOI:** 10.1016/S1473-3099(19)30695-4

**Published:** 2020-06

**Authors:** David A Barr, Joseph M Lewis, Nicholas Feasey, Charlotte Schutz, Andrew D Kerkhoff, Shevin T Jacob, Ben Andrews, Paul Kelly, Shabir Lakhi, Levy Muchemwa, Helio A Bacha, David J Hadad, Richard Bedell, Monique van Lettow, Rony Zachariah, John A Crump, David Alland, Elizabeth L Corbett, Krishnamoorthy Gopinath, Sarman Singh, Rulan Griesel, Gary Maartens, Marc Mendelson, Amy M Ward, Christopher M Parry, Elizabeth A Talbot, Patricia Munseri, Susan E Dorman, Neil Martinson, Maunank Shah, Kevin Cain, Charles M Heilig, Jay K Varma, Anne von Gottberg, Leonard Sacks, Douglas Wilson, S Bertel Squire, David G Lalloo, Gerry Davies, Graeme Meintjes

**Affiliations:** a Institute of Infection and Global Health, University of Liverpool, Liverpool, UK; b Wellcome Centre for Infectious Diseases Research in Africa, University of Cape Town, Cape Town, South Africa; c Institute of Infectious Disease and Molecular Medicine, University of Cape Town, Cape Town, South Africa; d Division of Infectious Diseases and HIV Medicine, University of Cape Town, Cape Town, South Africa; e Department of Medicine, University of Cape Town, Cape Town, South Africa; f Liverpool School of Tropical Medicine, Liverpool, UK; g Malawi-Liverpool-Wellcome Clinical Research Programme, Queen Elizabeth Central Hospital, College of Medicine, Blantyre, Malawi; h Division of HIV, Infectious Diseases, and Global Medicine at Zuckerberg San Francisco General Hospital and Trauma Center, Department of Medicine, University of California San Francisco, San Francisco, CA, USA; i Institute for Global Health, Vanderbilt University School of Medicine, Nashville, TN, USA; j Blizard Institute, Barts and London School of Medicine, Queen Mary University of London, London, UK; k Department of Internal Medicine, University of Zambia School of Medicine and University Teaching Hospital, Lusaka, Zambia; l Defence Force School of Health Sciences, Lusaka, Zambia; m Instituto de Infectologia Emilio Ribas, São Paulo, Brazil; n Universidade Federal do Espirito Santo, Centro de Ciêncicas da Saúde, Departamento de Clinica Médica, Vitoria, Brazil; o Dignitas International, Zomba, Malawi; p Division of Global Health, University of British Columbia, Vancouver, BC, Canada; q Dalla Lana School of Public Health, University of Toronto, Toronto, ON, Canada; r Medecins Sans Frontieres, Operational Centre Brussels, Brussels, Belgium; s Centre for International Health, University of Otago, Dunedin, New Zealand; t Division of Infectious Diseases and International Health, Duke University Medical Center, Durham, NC, USA; u Kilimanjaro Christian Medical Centre, Moshi, Tanzania; v Division of Infectious Disease, Department of Medicine, Rutgers-New Jersey Medical School, Newark, NJ, USA; w London School of Hygiene and Tropical Medicine, London, UK; x Max Planck Institute for Infection Biology, Berlin, Germany; y Division of Clinical Microbiology and Molecular Medicine, All India Institute of Medical Sciences, New Delhi, India; z School of Tropical Medicine and Global Health, University of Nagasaki, Nagasaki, Japan; aa Infectious Disease and International Health, Dartmouth Medical School, Hanover, NH, USA; ab Department of Internal Medicine, Muhimbili University of Health and Allied Sciences, Dar es Salaam, Tanzania; ac Johns Hopkins University Centre for TB Research, Johns Hopkins School of Medicine, Baltimore, MD, USA; ad Perinatal HIV Research Unit, South African Medical Research Council Soweto Matlosana Collaborating Centre for HIV/AIDS and TB, Centre of Excellence for Biomedical TB Research, University of the Witwatersrand, Johannesburg, South Africa; ae School of Pathology, Faculty of Health Sciences, University of the Witwatersrand, Johannesburg, South Africa; af Center for Surveillance, Epidemiology, and Laboratory Services, Atlanta, GA, USA; ag US Centers for Disease Control and Prevention, Atlanta, GA, USA; ah Centre for Respiratory Diseases and Meningitis, National Institute for Communicable Diseases of the National Health Laboratory Service, Johannesburg, South Africa; ai Office of Medical Policy, Center for Drug Evaluation and Research, US Food and Drug Administration, Silver Spring, MD, USA; aj Department of Internal Medicine, Edendale Hospital, University of KwaZulu-Natal, Pietermaritzburg, South Africa

## Abstract

**Background:**

The clinical and epidemiological significance of HIV-associated *Mycobacterium tuberculosis* bloodstream infection (BSI) is incompletely understood. We hypothesised that *M tuberculosis* BSI prevalence has been underestimated, that it independently predicts death, and that sputum Xpert MTB/RIF has suboptimal diagnostic yield for *M tuberculosis* BSI.

**Methods:**

We did a systematic review and individual patient data (IPD) meta-analysis of studies performing routine mycobacterial blood culture in a prospectively defined patient population of people with HIV aged 13 years or older. Studies were identified through searching PubMed and Scopus up to Nov 10, 2018, without language or date restrictions and through manual review of reference lists. Risk of bias in the included studies was assessed with an adapted QUADAS-2 framework. IPD were requested for all identified studies and subject to harmonised inclusion criteria: age 13 years or older, HIV positivity, available CD4 cell count, a valid mycobacterial blood culture result (excluding patients with missing data from lost or contaminated blood cultures), and meeting WHO definitions for suspected tuberculosis (presence of screening symptom). Predicted probabilities of *M tuberculosis* BSI from mixed-effects modelling were used to estimate prevalence. Estimates of diagnostic yield of sputum testing with Xpert (or culture if Xpert was unavailable) and of urine lipoarabinomannan (LAM) testing for *M tuberculosis* BSI were obtained by two-level random-effect meta-analysis. Estimates of mortality associated with *M tuberculosis* BSI were obtained by mixed-effect Cox proportional-hazard modelling and of effect of treatment delay on mortality by propensity-score analysis. This study is registered with PROSPERO, number 42016050022.

**Findings:**

We identified 23 datasets for inclusion (20 published and three unpublished at time of search) and obtained IPD from 20, representing 96·2% of eligible IPD. Risk of bias for the included studies was assessed to be generally low except for on the patient selection domain, which was moderate in most studies. 5751 patients met harmonised IPD-level inclusion criteria. Technical factors such as number of blood cultures done, timing of blood cultures relative to blood sampling, and patient factors such as inpatient setting and CD4 cell count, explained significant heterogeneity between primary studies. The predicted probability of *M tuberculosis* BSI in hospital inpatients with HIV-associated tuberculosis, WHO danger signs, and a CD4 count of 76 cells per μL (the median for the cohort) was 45% (95% CI 38–52). The diagnostic yield of sputum in patients with *M tuberculosis* BSI was 77% (95% CI 63–87), increasing to 89% (80–94) when combined with urine LAM testing. Presence of *M tuberculosis* BSI compared with its absence in patients with HIV-associated tuberculosis increased risk of death before 30 days (adjusted hazard ratio 2·48, 95% CI 2·05–3·08) but not after 30 days (1·25, 0·84–2·49). In a propensity-score matched cohort of participants with HIV-associated tuberculosis (n=630), mortality increased in patients with *M tuberculosis* BSI who had a delay in anti-tuberculosis treatment of longer than 4 days compared with those who had no delay (odds ratio 3·15, 95% CI 1·16–8·84).

**Interpretation:**

In critically ill adults with HIV-tuberculosis, *M tuberculosis* BSI is a frequent manifestation of tuberculosis and predicts mortality within 30 days. Improved diagnostic yield in patients with *M tuberculosis* BSI could be achieved through combined use of sputum Xpert and urine LAM. Anti-tuberculosis treatment delay might increase the risk of mortality in these patients.

**Funding:**

This study was supported by 10.13039/100010269Wellcome fellowships 109105Z/15/A and 105165/Z/14/A.

**Research in context**

**Evidence before this study**

We searched PubMed, Scopus, and the Cochrane Database of Systematic Reviews using combinations of the search terms “blood stream infection”, “bacter?emia”, “septic?emia”, “sepsis”, “tuberculosis”, “TB”, and “mycobacter?emia” up to Sept 15, 2019, without date or language restrictions for studies that have systematically summarised the available data on prevalence and mortality of HIV-associated tuberculosis bloodstream infection (BSI). We identified one aggregate meta-analysis of HIV-associated tuberculosis BSI, which reported that *Mycobacterium tuberculosis* is a common cause of BSI in adults with HIV infection. Two other aggregate data meta-analyses highlighted the high prevalence of tuberculosis as a cause of sepsis and community-acquired bloodstream infection in sub-Saharan Africa. These analyses showed substantial between-study heterogeneity, which was unexplained by study-level confounders. Although some published cohort studies have linked positive tuberculosis blood culture with increased risk of death, others have found no significant association; the identified meta-analyses have not reported pooled mortality associations adjusted for individual patient characteristics. Consequently, uncertainty exists about the clinical and epidemiological importance of *M tuberculosis* BSI.

**Added value of this study**

To address these uncertainties, we did an individual patient data (IPD) meta-analysis of health-care facility-based studies that did routine tuberculosis blood cultures on samples taken from adults with HIV infection. Correcting for individual patient characteristics, we were able to explain substantial variation in the probability of *M tuberculosis* BSI and found prevalence to be higher than previously reported, particularly in hospital inpatients with HIV-associated tuberculosis and WHO danger signs. We showed that *M tuberculosis* BSI in patients with HIV-associated tuberculosis was independently associated with death before 30 days. We found substantial heterogeneity in diagnostic yield of sputum Xpert and urine lipoarabinomannan (LAM) tests in patients with HIV-associated *M tuberculosis* BSI, which could in part be explained by a lower probability of obtaining samples from critically ill patients rather than by poor test diagnostic sensitivity. As seen for non-tuberculosis sepsis in high-income settings, we found that a delay in anti-tuberculosis treatment of more than 4 days is associated with 30-day or inpatient mortality in patients with HIV-associated *M tuberculosis* BSI.

**Implications of all the available evidence**

Tuberculosis in critically ill people with HIV is frequently a bloodstream infection, and tuberculosis bacteraemia is a common and important predictor of 30-day mortality. As with other causes of bacterial sepsis, providing prompt effective antimicrobial therapy reduce risk of mortality from *M tuberculosis* BSI. Urine LAM testing should be routinely added to first-line diagnostic testing of sputum in HIV-positive inpatients with suspected tuberculosis and with at least one WHO danger sign. Patients with *M tuberculosis* BSI are an important population in whom WHO management guidelines for seriously ill people with HIV suspected of having tuberculosis should be validated and warrant specific focus in the road map for future research and global response to sepsis. Interventional trials are urgently required to establish an evidence base for mortality reduction in patients with *M tuberculosis* BSI.

## Introduction

In settings with high HIV and tuberculosis burden, *Mycobacterium tuberculosis* bloodstream infection (BSI) might be common. When sought, tuberculosis is the most frequently identified community-acquired BSI in admitted to hospital adults in sub-Saharan Africa^1^ and in adult sepsis cohorts recruited in high-HIV burden settings.^2^, ^3^, ^4^ The high frequency of multiorgan, notably spleen, involvement in post mortems of patients with HIV-associated tuberculosis^5^ is consistent with active bloodstream dissemination being near universal in fatal cases.

Most settings with generalised HIV epidemics have no access to mycobacterial blood culture. Even where available, an average 3-week delay between culture and detection,^3^, ^6^, ^7^, ^8^ combined with high early mortality, means that tuberculosis blood culture has limited diagnostic value. Unlike other bacteraemic pathogens, no specific evidence base exists for treating patients with *M tuberculosis* BSI.

The true prevalence of *M tuberculosis* BSI is unknown; substantial unexplained heterogeneity exists in aggregate data meta-analysis.^9^ This heterogeneity might be explained by differences between studies in inclusion criteria, clinical factors such as CD4 cell count, or technical factors such as number of blood cultures.^10^ The diagnostic performance of tuberculosis rapid tests in HIV-associated tuberculosis is variable;^11^ the relative utility of sputum Xpert MTB/RIF (Cepheid, Sunnyvale, CA, USA) and urine lipoarabinomannan (LAM) depends on disease severity^12^ and might differ between patients with *M tuberculosis* BSI and those with HIV-associated tuberculosis who are less critically ill.^13^ Furthermore, although some studies have linked mycobacteraemia to high risk of death in HIV-associated tuberculosis,^3^, ^8^, ^14^ others found no significant independent association,^15^, ^16^, ^17^ which might be the result of underpowering or bias in application of the tuberculosis blood culture reference test.

WHO guidelines on HIV-associated tuberculosis do not directly address *M tuberculosis* BSI, but do refer to disseminated tuberculosis, described as disease not limited to one site. Additionally, WHO gives guidance, largely based on expert opinion, for managing seriously ill people living with HIV and suspected of having tuberculosis (panel).^18^ We hypothesised that a substantial proportion of inpatients with HIV-associated tuberculosis who are seriously ill have *M tuberculosis* BSI, and that they represent a group at particularly high risk of death, especially if effective treatment is delayed. If our hypothesis is correct, patients with *M tuberculosis* BSI would be an important population in whom the WHO algorithm should be validated.

Panel **Summary of WHO guidance for managing seriously ill people with HIV suspected of having tuberculosis18**

• Tuberculosis should be suspected if cough, fever, night sweats, or weight loss are present
• Patients are seriously ill if any of the following danger signs are present: respiratory rate less than 30 breaths per min, temperature below 39°C, heart rate higher than 120 beats per min, and inability to walk unaided
• In all cases, patients should be admitted to hospital and begin parenteral antibiotic treatment for bacterial infections
• Xpert MTB/RIF testing of sputum and extrapulmonary samples should be done if extrapulmonary tuberculosis is suspected
• If Xpert MTB/RIF test results are negative or the test is not available and there is no clinical improvement after 3–5 days, presumptive tuberculosis therapy should be started
• Urine lateral flow lipoarabinomannan test can be used regardless of CD4 cell count

We did an individual patient data (IPD) meta-analysis, allowing for harmonised individual patient inclusion criteria and adjustment for individual-level variables, to address four questions. First, what is the prevalence of *M tuberculosis* BSI in adult inpatients with HIV-associated tuberculosis who are seriously ill (having at least one WHO danger sign; panel)? Second, what is the diagnostic yield of sputum Xpert and urine LAM in patients with *M tuberculosis* BSI? Third, what is the mortality risk associated with having a positive tuberculosis blood culture? Finally, what is the effect on mortality of delaying anti-tuberculosis treatment by 3–5 days, as per the WHO algorithm, in patients with *M tuberculosis* BSI?

## Methods

### Search strategy and selection criteria

For this IPD meta-analysis, we searched databases for studies wherein mycobacterial blood culture was done in a prospectively defined patient population that included people living with HIV aged 13 years or older. We excluded studies in which CD4 cell count was not measured.

We searched PubMed and Scopus from database inception up to Nov 10, 2018, with no language or publication period restriction using the search terms ([“Blood stream infection” OR “BSI” OR “bacter?emia” OR “septic?emia”] AND [“tuberculosis” OR “TB”] OR [“mycobacter?emia”]). We also searched reference lists and review articles identified in the primary search. We contacted researchers with interest in HIV-associated tuberculosis to identify any unpublished cohorts. Two independent reviewers (DAB and JML) selected abstracts and obtained full texts of potentially eligible studies. Full texts were also reviewed independently by DAB and JML, with disagreements resolved by consensus after clarification of method details with the primary authors.

### Data extraction and processing

We asked the original investigators of the identified studies to provide primary data or meta-data in the event of unclear data coding. Prespecified variables (appendix pp 2–3) were extracted and standardised. Original case definitions for final tuberculosis diagnosis and microbiological identification standards were accepted.

We classified primary studies by inclusion criteria (suspected tuberculosis, inpatient, outpatient, patients with sepsis, febrile patients) and setting (tertiary care hospital, secondary care hospital, outpatient clinics, HIV testing service, specialist ifectious diseases centres). We assessed risk of bias in primary studies with an adapted QUADAS-2 framework that was informed by a survey sent to primary study authors (appendix pp 4–5). Two authors (DAB and JML) used the survey data to assess risk of bias as low, moderate, or high across five domains (patient selection, reference test, recording of co-factors [ie, covariates to be included in model], index test, and mortality outcome), with disagreements resolved by consensus.

Harmonised inclusion criteria were applied to IPD: age 13 years or older, HIV positivity, available CD4 cell count, a valid mycobacterial blood culture result (excluding patients with missing data from lost or contaminated blood cultures), and WHO tuberculosis screening symptoms.^18^ All patients included met WHO definitions for suspected tuberculosis.^18^

### Estimating the prevalence of *M tuberculosis* BSI in seriously ill patients with HIV

*M tuberculosis* BSI prevalence was assessed with mixed-effects logistic regression using lme4 package in R.^19^ Random intercept by primary study and fixed effects for a-priori-specified variables were added to the model in an arbitrary, prespecified order (appendix p 10) using the raw (unimputed) datasets. Continuous variables were standardised to a mean of 0 and a SD of 1. Each nested model was compared with the likelihood–ratio test (LRT) to a null model containing only a random effect by study on the intercept and the preceding model, giving p values reported as LRT_null_ and LRT_preceding_, respectively. The effect of the models on heterogeneity was assessed with random effects variance (τ^2^) and proportion of residual individual variance attributable to random effects (variance partition coefficient).^20^, ^21^ Variance explained by fixed effects (R^2^_marginal_) and by model containing fixed and random effects (R^2^_conditional_) was calculated using r.squaredGLMM function in MuMIn R package.^22^, ^23^ The importance of clustering by primary dataset was further assessed by calculating the additional area under the receiver operating characteristic curve (AUC; within sample discrimination) obtained from including random effects by dataset (appendix p 6).^20^

All variables with an LRT_preceding_ p value of less than 0·01 were included in the final model, and the predicted probability of positive tuberculosis blood culture from this model was used to simulate the population prevalence of *M tuberculosis* BSI for given levels of the relevant fixed-effects (eg, assuming two blood cultures were done before anti-tuberculosis treatment was started and at a specific CD4 cell count). An overall mean prevalence of *M tuberculosis* BSI in an average study was calculated (ie, with a random effect of 0), as well as for each primary study (including associated random effects) to visualise residual heterogeneity in *M tuberculosis* BSI probability after adjusting for IPD-level covariates.

We did multiple imputation for systematically missing data (ie, variables missing for an entire study dataset) and sporadically missing data (ie, variables that were available for a given dataset but were missing for individuals) using generalised linear mixed models (GLMMs) to account for clustering by primary study, using the hmi package in R.^24^ Missing observations in each variable from the set (heart rate, respiratory rate, temperature, ability to walk unaided, early mortality, patient setting, age, sex, CD4 cell count, *M tuberculosis* BSI, and haemoglobin) were imputed with all the other variables in the set as predictors using logistic or linear GLMMs, as appropriate. Five imputations were done, resulting in five complete versions of the data.

Mean predicted prevalence values and 95% CIs were calculated from pooled bootstraps, with resampling stratified by primary study. 1000 replicates from each of the five imputed versions of the data were pooled and CIs derived from their quantiles.^25^ To assess the effect of bias on our results, a sensitivity analysis was done whereby any study with high or unknown risk of bias in any in any domain was excluded and this bootstrap procedure repeated.

Finally, a 95% prediction interval for mean prevalence of *M tuberculosis* BSI in a new unobserved study was estimated from 1000 simulations in each of the five imputed versions of the data using bootMer in lme4,^19^ capturing uncertainty in parameter estimates, random variation between studies, conditional variation in the binary outcome, and variance from imputation of missing data.^26^

### Estimating the utility of rapid-diagnostic tools in patients with *M tuberculosis* BSI

To assess the utility of rapid diagnostics to identify tuberculosis in patients with *M tuberculosis* BSI, we defined sputum diagnostic yield as the proportion of patients with *M tuberculosis* BSI who had a positive sputum test result, using an aggregate sputum variable of Xpert or *M tuberculosis* culture as a surrogate if Xpert was unavailable (which assumes that Xpert would be positive for all patients with a positive sputum culture); urine LAM diagnostic yield as the proportion of patients with *M tuberculosis* BSI who had a positive urine LAM test; and composite diagnostic yield as the proportion of patients with *M tuberculosis* BSI who had either test positive. We restricted this analysis to studies that collected IPD on results of sputum Xpert (or culture) or urine LAM. We did not do protocol-specified analyses of chest radiology and sputum microscopy because of insufficient data. Pooled estimates of diagnostic yield were obtained by two-level random-effect meta-analysis using a normal-binomial GLMM method in the R packages meta and lme4.^19^, ^27^ We explored heterogeneity using meta-regression on prespecified study-level covariates, assessing the role of covariates by LRT nested models. Factors associated with availability of sputum or urine were assessed in univariable mixed-effect logistic regression. Because absence of a diagnostic test was thought to be a probable determinant of diagnostic yield, we analysed unimputed data, and the proportion of participants with an available test result was included as a covariate.

### Estimating mortality associated with *M tuberculosis* BSI

To assess the independent mortality risk of *M tuberculosis* BSI in patients with HIV-associated tuberculosis, we constructed mixed-effect Cox proportional hazard models using survival package in R,^28^ with random intercept by study and a-priori specified fixed effects: age, sex, CD4 cell count, inpatient versus outpatient, presence of WHO danger signs, on antiretroviral therapy (ART) at baseline, and *M tuberculosis* BSI (defined as positive blood culture). The proportional hazards assumption was checked by χ^2^ test of a non-zero slope of scaled Schoenfeld residuals against time. Missing data were imputed using the strategy described for estimating the prevalence of *M tuberculosis* BSI; CIs on model parameters were constructed by pooling 1000 non-parametric cluster bootstrap replicates from five imputed datasets as described for the estimate of *M tuberculosis* BSI prevalence. Unadjusted hazard ratios (HRs) for all the fixed-effect variables were calculated with models that included only the variable of interest and a random intercept by study; adjusted HRs were calculated with a model that included all the fixed-effect variables along with a random intercept by study. Cox regression was used in preference to the protocol-specified logistic regression because observations on date of death were more complete than anticipated.

### Estimating the effect of anti-tuberculosis treatment delay on mortality

Finally, in an unplanned analysis, we explored the association between time to anti-tuberculosis treatment and mortality in patients with *M tuberculosis* BSI. To reflect the 3–5 day observation period in the WHO algorithm (panel), treatment delay was defined as more than 4 days between blood culture collection and start of anti-tuberculosis treatment; other cutoffs were explored by sensitivity analysis. Mortality was defined as death before discharge from hospital or by 30-days' follow-up. A high proportion of observations for the variable start date of anti-tuberculosis treatment were unavailable (1059 [43%] of 2460 participants; appendix p 22); we thought these to be most likely to be missing completely at random and did a complete case analysis; patients without a final tuberculosis diagnosis, without complete observations, or starting anti-tuberculosis treatment 24 h or more before blood culture were excluded. We calculated a propensity score for anti-tuberculosis treatment delay using logistic regression with the variables age, CD4 cell count, one or more WHO danger sign (panel), mycobacterial blood culture result, and primary study. Patients without anti-tuberculosis treatment delay were matched 2:1 with patients with anti-tuberculosis treatment delay by propensity score nearest neighbour matching on logit distance, without calliper restrictions. We assessed the association between anti-tuberculosis treatment delay and mortality in the whole matched cohort and in prespecified subgroups (including only patients with *M tuberculosis* BSI, danger signs, or CD4 counts <100 cells per μL) using Fisher's exact test.

All analyses were done in R version 1.40.0. The meta-analysis protocol is registered with PROSPERO (42016050022).

### Role of the funding source

The funder had no role in the study design, data collection, data analysis, data interpretation, or writing of this report. The corresponding author had full access to the all the data in the study and had final responsibility for the decision to submit for publication.

## Results

**Figure 1:**
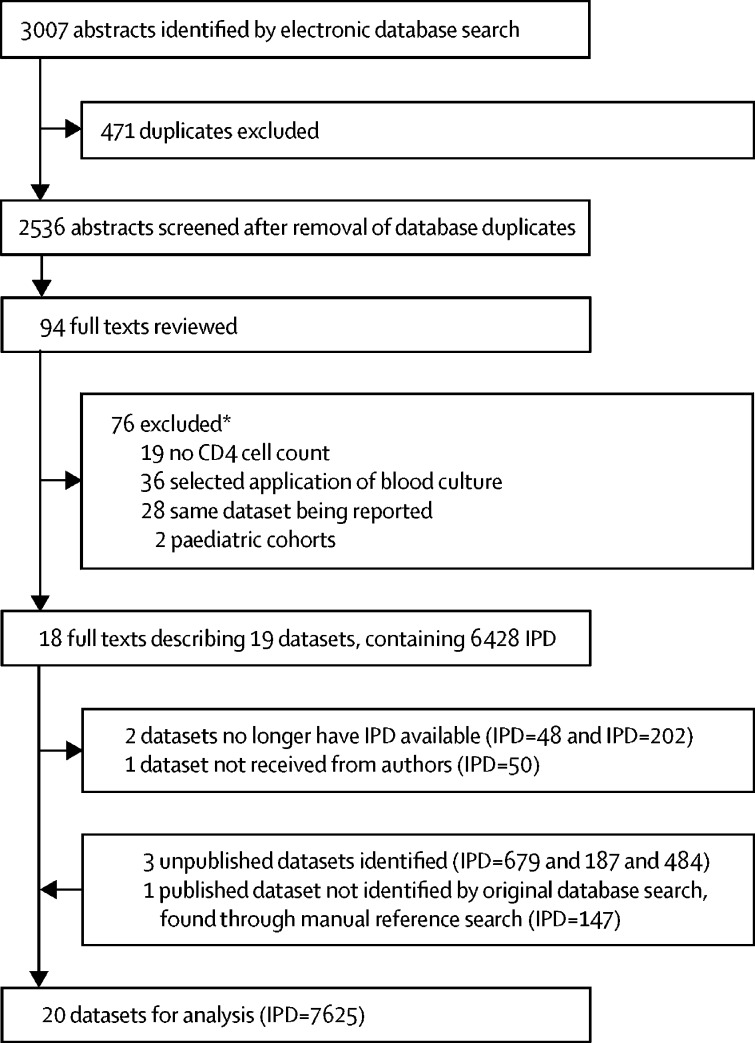
Study selection IPD=individual patient data. *Some studies met more than one exclusion criterion.

The database search identified 19 datasets for inclusion; four additional datasets were identified from other sources (three were unpublished and one was missed by database search terms; figure 1). Responses were obtained from all primary study authors, and IPD was available for 20 datasets, representing 96·2% (7625 of 7926) of the sought IPD (IPD was lost for two datasets and one dataset was not received; figure 1). Generally, risk of bias was assessed to be low, except for on one domain, patient selection, which was moderate for most studies (appendix pp 4–5, 7). Application of the harmonised IPD-level inclusion criteria left 5751 patients for analyses. Characteristics of these patients and missing data by study are shown in the appendix (appendix pp 8–9). No data from high-income settings met inclusion criteria; 74% of included patients were recruited in sub-Saharan Africa.

Compared with the prevalence of *M tuberculosis* BSI reported in the original publications (mean prevalence weighted by study n, 9·76%; coefficient of variation 61·3% for reported prevalences; table 1) the proportion of patients with *M tuberculosis* BSI after application of harmonised IPD inclusion criteria was higher but no less heterogeneous (prevalence recorded *M tuberculosis* BSI in all included IPD, 14·4%; coefficient of variation 61·9%; appendix p 9). Mixed-effect logistic regression models (appendix p 10) showed that six of eight a-priori selected variables were associated with *M tuberculosis* BSI (CD4 cell count, presence of danger signs, hospitalisation status, receiving tuberculosis treatment before blood culture, number of blood cultures done, and final diagnosis of tuberculosis) and two were non-significant (ART status and year of recruitment). Compared with the model containing no fixed effects, inclusion of the six significant predictor variables in the model reduced heterogeneity between datasets (τ^2^ reduced from 0·79 to 0·49, variance partition coefficient reduced from 0·19 to 0·13), explained substantial total variance (R^2^_conditional_ increased from 0·19 to 0·73), improved within-sample predictive accuracy (AUC increased from 0·75 to 0·91), and reduced the importance of random effects for predictive accuracy (additional AUC reduced from 0·25 to 0·01).

**Table 1:** Primary datasets

	**Country or region**	**Source**	**IPD data received?**	**Site**	**Design**	**Dates**	**Primary study population**	*M tuberculosis* BSI prevalence reported (%)*	**Number of patients with IPD available**	**Number of patients who met inclusion criteria**†
Grinsztejn et al (1997)	Brazil	Database search	No	Three specialist infectious disease centres	Cohort	1992–94	Inpatients with suspected tuberculosis	38·0	NA	NA
Bacha et al (2004)	Brazil	Database search	Yes	Tertiary care hospital	Cohort	2001–02	Inpatients with suspected tuberculosis	29·5	53	44
Gopinath et al (2008)	India	Database search	Yes	Tertiary care hospital	Cohort	2005–06	Inpatients with suspected tuberculosis	30·8	52	36
Vugia et al (1993)	Ivory Coast	Database search	Not available	Tertiary care hospital	Cohort	1991	Febrile inpatients	4·0	NA	NA
Gilks et al (1995)	Kenya	Database search	Not available	Tertiary care hospital	Cohort	1992	Inpatients with suspected tuberculosis	22·9	NA	NA
Bedell et al (2012)	Malawi	Database search	Yes	Outpatient clinics	Cohort	2010	Outpatients with suspected tuberculosis	2·3	469	411
Feasey et al (2013)	Malawi	Database search	Yes	Secondary care hospital	Cohort	NA	Febrile inpatients	8·7	104	90
Von Gottberg et al (2001)	South Africa	Database search	Yes	Tertiary care hospital	Cohort	1998	Inpatients with suspected tuberculosis	22·5	45	44
Wilson et al (2006)	South Africa	Manual reference search	Yes	Secondary care hospital	Cohort	2002	Inpatients and outpatients with suspected tuberculosis	24·5	147	141
Shah et al (2009)	South Africa	Database search	Yes	Tertiary and secondary care hospitals	Cohort	NA	Inpatients with suspected tuberculosis	8·6	498	264
Nakiyingi et al (2014)	South Africa	Database search	Yes	Secondary care hospitals and outpatient clinics	Cohort	2011	Inpatients and outpatients with suspected tuberculosis	9·5	513	483
Lawn et al (2015)	South Africa	Database search	Yes	Secondary care hospital	Cohort	2012–13	Inpatients	9·6	427	338
Griesel et al (2017)	South Africa	Personal contact	Yes	Secondary care hospitals	Cohort	2011–14	Inpatients with suspected tuberculosis	23·6	484	444
Schutz et al (2018)	South Africa	Personal contact	Yes	Secondary care hospital	Cohort	2014–17	Inpatients with suspected tuberculosis	NA	679	615
Varma et al (2010)	Southeastern Asia	Database search	Yes	HIV testing service outpatient clinic	Cohort	2006–08	Outpatients	1·8	2009	1338
Munseri et al (2011)	Tanzania	Database search	Yes	Secondary and tertiary care hospitals	RCT	2007–08	Inpatients with suspected tuberculosis	15·9	258	230
Crump et al (2012)	Tanzania	Database search	Yes	Tertiary care hospitals	Cohort	2006–10	Febrile inpatients	5·7	411	145
Jacob et al (2009)	Uganda	Database search	Yes	Tertiary care hospitals	Cohort	2006	Inpatients with sepsis	22·1	150	98
Jacob et al (2013)	Uganda	Database search	Yes	Tertiary care hospitals	Cohort	2008–09	Inpatients with sepsis	23·4	427	315
Nakiyingi et al (2014)	Uganda	Database search	Yes	Secondary care hospitals and outpatient clinics	Cohort	2011	Inpatients and outpatients with suspected tuberculosis	15·6	524	479
Louie et al (2004)	Vietnam	Database search	Yes	Tertiary care hospital	Cohort	2000	Inpatients	12·3	100	61
Andrews et al (2014)	Zambia	Database search	Yes	Tertiary care hospital	RCT	2012	Inpatients with sepsis	37·8	88	58
Andrews et al (2017)	Zambia	Personal contact	Yes	Tertiary care hospital	RCT	2012–13	Inpatients with sepsis	20·6	187	117

All studies did mycobacterial blood culture in prospectively defined patient populations of people with HIV aged 13 years or older and measured CD4 cell count. Full citations in are in the appendix, pp 25–26. BSI=bloodstream infection. IPD=individual patient data. NA=not available. RCT=randomised controlled trial.

* Disaggregated HIV-positive sample if available.

† IPD-level inclusion criteria were age 13 years or older, confirmed HIV infection, available CD4 count, at least one valid mycobacterial blood culture result (excluding patients with missing data—eg, from lost or contaminated blood cultures), and at least one WHO tuberculosis screening symptom.

**Figure 2:**
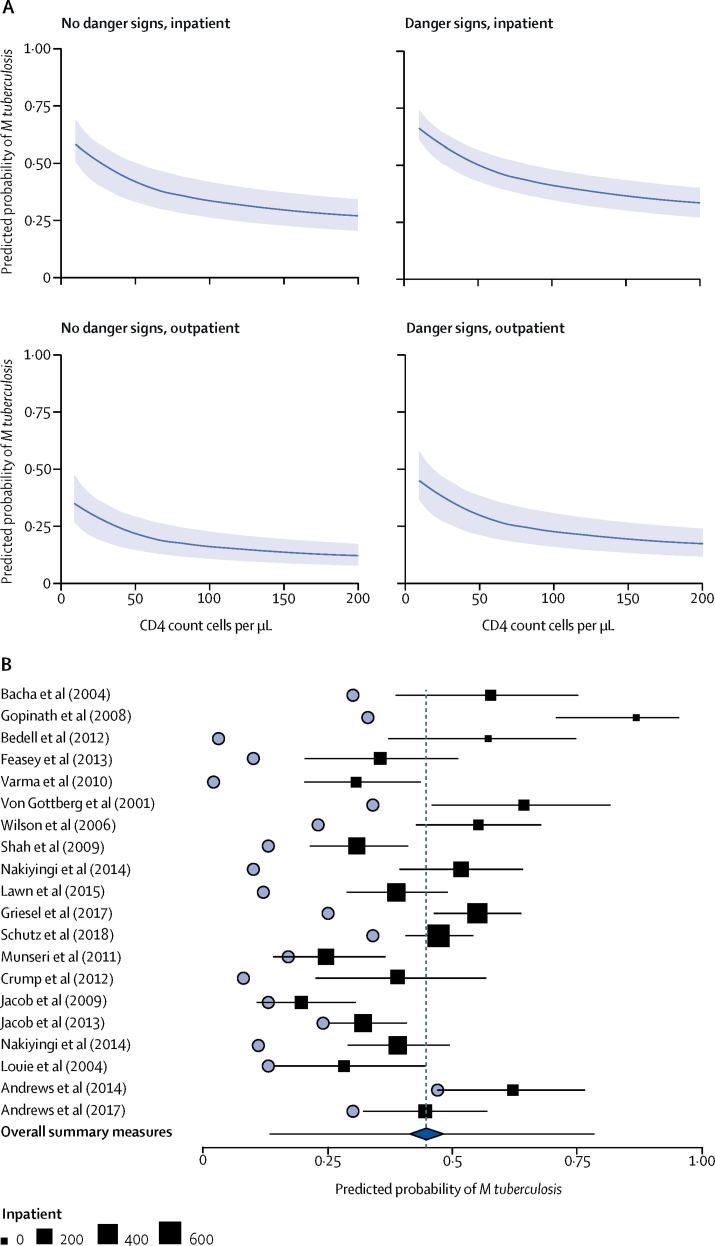
Predicted probability of *Mycobacterium tuberculosis* BSI in patients with HIV-associated tuberculosis All predictions assume that two tuberculosis blood cultures had been done before the start of anti-tuberculous therapy. (A) Simulated probability of positive tuberculosis blood culture for people with HIV diagnosed with tuberculosis at varying covariate levels; the solid line represents the mean predicted probabilities and the shading represents the 95% CI. (B) Predicted probability (squares) with 95% CI (whiskers) of a positive tuberculosis blood culture in inpatients with HIV-associated tuberculosis and WHO danger signs, with a CD4 count of 76 cells per μL (the median across datasets); the size of the square is proportional to the number of hospital inpatients in each study. The vertical dashed line indicates the population mean (all datasets combined) and the blue diamond the 95% CI around that mean; the 95% prediction interval for the mean predicted probability of *M tuberculosis* BSI in a new, unobserved dataset is shown by whiskers around the diamond. Also shown for comparison are the tuberculosis blood culture positivity rates originally reported for each primary study (blue circles). BSI=bloodstream infection.

The final model, following imputation of missing data, was used to simulate the prevalence of *M tuberculosis* BSI in patients diagnosed with HIV-associated tuberculosis when two blood cultures have been collected before the start of anti-tuberculosis treatment. Inpatients with one or more WHO danger signs and CD4 counts lower than 100 cells per μL had the highest predicted probability of *M tuberculosis* BSI (figure 2A). For hospital inpatients with WHO danger signs and CD4 counts of 76 cells per μL (the median for inpatients, IQR 24–185), the population mean predicted probability of *M tuberculosis* BSI (ie, across all datasets) was 0·45 (95% CI 0·38–0·52, figure 2B). The 95% prediction interval for the mean probability of *M tuberculosis* BSI in a new study was 0·14–0·78 (figure 2B), which was wider, as would be expected, than the 95% CI of the population mean predicted probability, reflecting remaining between-study heterogeneity despite the inclusion of covariates (figure 2). In a sensitivity analysis excluding studies with high or unknown risk of bias, the estimated prevalence of *M tuberculosis* BSI in patients with HIV-associated tuberculosis was 0·38 (95% CI 0·31–0·41), similar to the main analysis but 7% lower, with CIs that overlapped the original estimates; the prediction interval for a new study mean was 0·18–0·59.

We estimated the diagnostic yield of sputum and urine LAM testing for patients with *M tuberculosis* BSI, using the aggregate sputum variable of Xpert or culture if Xpert was not available, and without imputation of missing data. In the 14 studies with sputum Xpert, 545 (84%) of 652 patients with *M tuberculosis* BSI had a valid sputum sample, of whom 464 (85%) tested positive. Of 554 patients with *M tuberculosis* BSI in the eight studies that did urine LAM testing, 422 (76%) had a valid urine LAM result, of whom 304 (72%) tested positive. In the six studies that did both sputum and urine LAM testing, 480 (98%) of 492 patients with *M tuberculosis* BSI had a valid sputum or urine LAM result, with 424 (88%) of 480 patients having at least one positive test. Availability and results of diagnostic testing stratified by study is shown in the appendix (pp 11–12). All studies performing Xpert used G4 cartridges, and all urine LAM testing used Alere Determine TB LAM Ag test (Alere, Waltham, MA, USA).

The pooled summary diagnostic yield of urine LAM was 52% (95% CI 35–69) and of sputum was 77% (63–87); the composite diagnostic yield (urine LAM plus sputum) was highest at 89% (95% CI 80–94, appendix p 13). There was significant heterogeneity across studies (appendix p 13). Meta-regression showed that the proportion of patients with an available test result explained significant heterogeneity in the diagnostic yield of sputum and urine LAM (both p< 0·0001). Restricting the analysis to the four studies that did sputum Xpert (ie, excluding those that used culture as a surrogate) reduced the diagnostic yield of sputum and increased uncertainty (72%, 95% CI 30–94; appendix p 14). Inability to provide sputum and urine was associated with *M tuberculosis* BSI and death, along with several other markers of critical illness, in a univariable analysis of unimputed data (appendix p 15).

**Figure 3:**
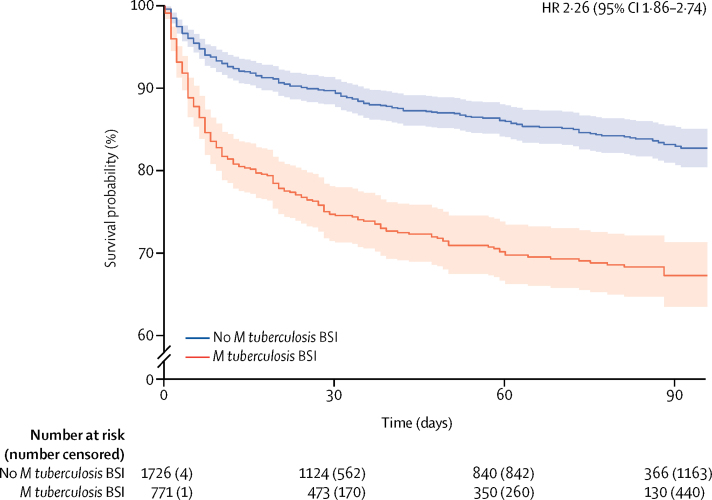
Pooled Kaplan-Meier curves (solid lines) and 95% CIs (shaded areas) for all patients with tuberculosis diagnosed by any means (n=2497), stratified by presence (red) or absence (blue) of *Mycobacterium tuberculosis* BSI Plot generated using a simple pooling of all data, without imputation of missing data. BSI=bloodstream infection.

We constructed mixed-effect Cox proportional hazards models to identify factors associated with mortality in patients diagnosed with tuberculosis (n=2497; characteristics shown in appendix p 16), following multiple imputation of missing data. Risk of mortality was higher in patients with HIV-associated tuberculosis with *M tuberculosis* BSI than in those without (figure 3). Because scaled Schoenfeld residuals of sex and presence of *M tuberculosis* BSI showed a significant interaction with time, coefficients were modelled separately for 0–30 days follow-up and 31–100 days follow-up. This decision (which was not included in our protocol) was made after inspection of the plot of the time-varying estimates of the coefficient of presence of *M tuberculosis* BSI against time (appendix p 17). *M tuberculosis* BSI significantly increased the risk of death before 30 days but not after 30 days in the final model (table 2). In the pooled analysis, urine LAM status was associated with mortality (OR 1·86, 95% CI 1·07–3·26), although there was evidence of between-study heterogeneity (appendix p 18). After adjusting for age, sex, WHO danger signs, CD4 cell count, and ART status in a post-hoc mixed-effect Cox proportional hazards model (equivalent to that used for *M tuberculosis* BSI), positive urine LAM was not significantly associated with mortality in patients with a diagnosis of HIV-associated tuberculosis (HR 1·24, 95% CI 0·86–2·36; appendix p 19).

**Table 2:** Risk of death in patients with a final diagnosis of tuberculosis (n=2497)

		**HR (95% CI)**	**Adjusted HR (95% CI)**
Outpatient (*vs* inpatient)		0·13 (0·00–0·23)	0·17 (0·07–0·33)
Age (per 5 years' increase)		1·11 (1·05–1·15)	1·12 (1·04–1·17)
Receiving antiretroviral therapy at baseline (yes *vs* no)		0·98 (0·54–1·37)	0·99 (0·56–1·62)
Presence of one or more WHO danger signs (yes *vs* no)		1·46 (0·95–2·03)	1·29 (0·80–1·63)
CD4 count (per 100 cells per μL increase)		0·81 (0·69–0·92)	0·83 (0·68–0·96)
Positive for *Mycobacterium tuberculosis* BSI*			
	During 0–30 days follow-up	2·82 (2·43–3·38)	2·48 (2·05–3·08)
	During 31–100 days follow-up	1·38 (0·95–2·76)	1·25 (0·84–2·49)
Sex (male *vs* female)*			
	During 0–30 days follow-up	1·45 (1·19–2·04)	1·27 (1·02–1·87)
	During 31–100 days follow-up	0·60 (0·41–1·22)	0·56 (0·39–1·13)

Unadjusted and adjusted HR from Cox proportional hazard model following imputation of missing data. Unadjusted HR includes a random-effect term by dataset. HR=hazard ratio. BSI=bloodstream infection.

* Scaled Schoenfeld residuals of sex and presence of *Mycobacterium tuberculosis* BSI showed a significant interaction with time; therefore, coefficients were modelled separately for 0–30 days and 31–100 days follow-up.

In a post-hoc, unimputed analysis, we examined the association between time to anti-tuberculosis treatment and early mortality (defined as 30-day or inpatient death). In patients with WHO danger signs with *M tuberculosis* BSI, early mortality was increased in participants who started anti-tuberculosis treatment before enrolment and those who started anti-tuberculosis treatment more than 4 days after enrolment compared with those who started treatment 0–4 days after enrolment (appendix p 20). We hypothesised that any causal relationship between time to anti-tuberculosis treatment and early mortality would be confounded by more urgent treatment in patients at higher risk of death, making shorter time to anti-tuberculosis treatment appear harmful. To adjust for this confounding, we did a propensity score analysis (causal assumptions are shown in a directed acyclic graph in appendix p 21). Of 1208 patients who met the inclusion criteria for this analysis, 630 (420 without and 210 with anti-tuberculosis treatment delay) were matched 2:1 by propensity score matching for anti-tuberculosis treatment delay (appendix pp 22–23). In patients with *M tuberculosis* BSI, 13 (27%) of 49 with anti-tuberculosis treatment delay died compared with ten (10%) of 98 who experienced no anti-tuberculosis treatment delay (OR 3·2, 95% CI 1·2 to 8·8; p=0·015, appendix p 20). This effect size was sensitive to the cutoff used to define anti-tuberculosis treatment delay, progressively reducing when treatment delay was classified as more than 3 days or more than 2 days (appendix p 24).

## Discussion

The results presented here indicate that *M tuberculosis* BSI is a common form of disease in hospital inpatients with advanced HIV-associated tuberculosis in low-income and middle-income settings. Previous estimates of *M tuberculosis* BSI prevalence are underestimates, with most previous studies relying on single blood cultures, not accounting for false-negative results from antimicrobial carry-over in plasma, and including patients with unobserved blood culture status (eg, due to contamination) in the denominator. Using modelling and simulation to account for these shortcomings, we estimated the *M tuberculosis* BSI prevalence to be 45% (95% CI 38–52) in patients with HIV-associated tuberculosis with WHO danger signs who have a median inpatient CD4 count of 76 cells per μL if two blood cultures are taken, with the prevalence increasing at lower CD4 cell counts. Substantial heterogeneity in reported *M tuberculosis* BSI prevalence is explained by these technical and clinical co-factors.

We found substantial heterogeneity in the diagnostic yield of rapid diagnostics in patients with *M tuberculosis* BSI, which was explained in part by the increased risk of unobtained samples in more critically ill patients. However, the combination of sputum and urine LAM testing gave a pooled diagnostic yield of 0·89 (95% CI 0·80–0·94) in studies where both tests were available. These studies often had dedicated staff to collect spontaneous or induced sputum, and so managed to obtain sputum from a high proportion of patients; such dedicated sputum collection might be unfeasable in routine practice. In a previous study,^29^ the addition of urine LAM testing to sputum Xpert reduced mortality in hospital inpatients with HIV with suspected tuberculosis who had a CD4 count of less than 100 cells per μL or severe anaemia, subgroups at highest risk of *M tuberculosis* BSI.

We found that *M tuberculosis* BSI independently predicted mortality before 30 days, with an adjusted HR of 2·5 (95% CI 2·1–3·1). Previous studies which found no association between mortality and *M tuberculosis* BSI might have been underpowered^16^, ^17^ or biased by selective application of the index test.^15^ By comparison, the association of urine LAM with mortality was less clear. Unavailability of urine for testing was associated with death, raising the possibility of missing-data bias, but imputation of missing urine LAM data did not affect the results. Urine LAM positivity might have a less direct causal relationship with death than positive tuberculosis blood culture, and therefore the association between tuberculosis blood culture and mortality might have been more robust to differences in comparator group case-mix between studies. Urine LAM might have less prognostic value in more critically ill cohorts, but strong prognostic value in a wider patient population.

We found associations between early mortality (30 day or inpatient) and very early or delayed tuberculosis treatment, an effect often seen in studies of non-mycobacterial sepsis, in which both groups with the shortest and those with the longest times to antimicrobial therapy have the highest risks of death,^30^, ^31^, ^32^ probably due to confounding by disease severity.^33^, ^34^ By accounting for this with propensity score matching, we found that delaying anti-tuberculosis therapy was associated with early mortality in patients with *M tuberculosis* BSI (OR 3·2, 95% CI 1·2–8·8).

A major limitation of this study is variation in inclusion criteria of the primary datasets, which we identified as the largest source of potential bias. Even after adjusting for technical and clinical factors, heterogeneity persisted between primary studies in prevalence estimates for *M tuberculosis* BSI. Subsequent adjustment for final diagnosis of tuberculosis resulted in the largest reduction in variance attributable to random effects between studies. Consequently, we limited our prevalence estimates to patients with HIV-associated tuberculosis and did not estimate prevalence in other protocol-specified subgroups (patients with suspected tuberculosis and patients with sepsis syndrome). Variations in study design and conduct could also explain heterogeneity in sputum diagnostic performance; two studies had explicit biases, exclusion of patients unable to produce sputum despite induction^35^ and exclusion of smear-positive patients.^36^ Datasets in which the primary study aim was to test the performance of diagnostics had the highest diagnostic yields, whereas cohorts recruited to explore mortality associations had lower yields. We relied on sputum culture as a surrogate for Xpert testing in studies without Xpert, which would have overestimated the sensitivity (and, therefore, the diagnostic yield) of sputum testing. Several studies had systematically missing data on cofactors. Missing data were multiply imputed with a method accounting for clustering by data set, and uncertainty associated with imputation accounted for in CIs; this imputation will have reduced the risk of missing data bias at the expense of greater imprecision. This method could not account for measurement error, which might explain the absence of an independent association between ART status and *M tuberculosis* BSI prevalence and death. We imputed five datasets; more might have been desirable but fitting the mixed-effect models on more imputed datasets would have been computationally infeasible in a reasonable timeframe. Another limitation is that 74% of included patients were recruited in sub-Saharan Africa; thus generalisation of findings should be done with care. We assessed risk of bias using a modified QUADAS-2 framework, but classifying risk of bias in observational IPD meta-analyses is a difficult task with no gold-standard tool available; bias might have been underestimated or overestimated. Finally, the analysis suggesting an association between mortality and anti-tuberculosis treatment delay was designed after protocol registration and data collection and is limited by sample size and the possibility of unmeasured confounding.

Our findings have several implications for clinical practice and guidelines for managing seriously ill patients with HIV-associated tuberculosis. When the WHO algorithm for managing seriously ill patients is being applied to patients with HIV-associated tuberculosis, it is in fact being applied to patients with the highest risk of *M tuberculosis* BSI. Therefore, the recommendations contained in the algorithm must be valid for patients with *M tuberculosis* BSI. Sputum testing with Xpert is the main rapid diagnostic step recommended in the WHO algorithm. There is substantial unexplained heterogeneity in the sensitivities of tuberculosis diagnostics in people with HIV.^11^, ^37^, ^38^, ^39^ Our results show that substantial variation in sample availability has an even greater effect on diagnostic yield in critically ill patients with *M tuberculosis* BSI. We found that the probability of obtaining sputum and urine was reduced in the sickest patients, a considerable concern for the WHO algorithm, as reliance on these rapid diagnostics might be delaying treatment in patients at greatest risk of death. Our results support routine use of both sputum Xpert and urine LAM in parallel for inpatients with danger signs to help offset this risk.

The WHO algorithm recommends delaying presumptive anti-tuberculosis treatment for 3–5 days when rapid tests are non-diagnostic. Our study raises a concern that this delay is associated with an increase in early mortality. Trials^40^, ^41^, ^42^ of presumptive tuberculosis therapy in people with HIV had negative results but recruited ambulant (without danger signs) outpatients, and so will have largely excluded patients with *M tuberculosis* BSI, in whom the benefit of early empirical therapy is most likely.

*M tuberculosis* BSI accounts for a disproportionate burden of disease in seriously ill people with HIV. Tuberculosis remains the major cause of in-hospital death in HIV-positive adults; *M tuberculosis* BSI is a major predictor of this mortality. The risks, benefits, and utility of rapid diagnostics and empirical therapy for patients with HIV-associated *M tuberculosis* BSI are different from those of patients with non-bacteraemic HIV-associated tuberculosis and require specific evidence. Trials of tuberculosis treatment in people living with HIV have almost exclusively recruited sputum smear-positive ambulant outpatients.^43^ Consequently, there is a striking scarcity of data supporting current management of HIV-associated *M tuberculosis* BSI. Pragmatic interventional studies assessing early empiric treatment or intensified therapy strategies for *M tuberculosis* BSI are needed.
