# Supplementary material for: Mycobacterium tuberculosis bloodstream infection prevalence, diagnosis, and mortality risk in seriously ill adults with HIV: a systematic review and meta-analysis of individual patient data
Source: Lancet Infect Dis. 2020 Jun;20(6):742–52. doi: 10.1016/S1473-3099(19)30695-4 (PMC7254058; doi:10.1016/S1473-3099(19)30695-4)
Supplement: Supplementary appendix [file mmc1.pdf]

THE LANCET
Infectious Diseases

Supplementary appendix

This appendix formed part of the original submission and has been peer reviewed. We post it as supplied by the authors.

Supplement to: Barr DA, Lewis JM, Feasey N, et al. *Mycobacterium tuberculosis* bloodstream infection prevalence, diagnosis, and mortality risk in seriously ill adults with HIV: a systematic review and meta-analysis of individual patient data. *Lancet Infect Dis* 2020; published online March 13. https://doi.org/10.1016/S1473-3099(19)30695-4.

Supplementary appendix

Table S1. Requested variables for IPD analysis.

Table S2. Bias assessment questionnaire adapted from QUADAS-2 tool.

Table S3 Measures of model fit and variance / heterogeneity explained in mixed-effect models predicting probability of MTB-BSI

Figure S1. Summary risk of bias assessment results by included primary dataset and domain.

Figure S2. Proportion data missing by variable and primary dataset.

Table S4. Characteristics of patients meeting IPD inclusion criteria by included primary dataset.

Table S5. Iterative mixed-effect models examining predictors of tuberculosis blood culture result and heterogeneity between datasets.

Table S6 Availability of TB diagnostic tests stratified by study.

Figure S3. Distribution of positive, negative and unavailable TB diagnostic testing stratified by study.

Figure S4. Diagnostic yield of rapid diagnostics in patients with MTB-BSI.

Figure S5. Sensitivity analysis exploring effect of composite sputum variable on diagnostic yield analyses.

Table S7. Associations of no sputum result available

Table S8. Associations of no urine-LAM result available

Table S9. Characteristics of patients with a final TB diagnosis in each primary study included in meta-analysis.

Figure S6. Time varying coefficient of presence of MTB -BSI fitted with natural spline with 4 degrees of freedom

Figure S7. Mortality-hazard associated with positive TB blood culture and urine-LAM in studies performing both tests.

Table S10. Adjusted hazard ratio of death in urine-LAM positive patients with diagnosis of TB.

Figure S8. Risk of death by treatment delay and patient group: raw data and propensity score analysis.

Figure S9. Directed Acyclic Graph (DAG) explicating assumptions made about causal structure for propensity score analysis of effect treatment delay on mortality.

Figure S10. Patient inclusion in treatment delay analysis.

Figure S11. Distribution of propensity score by observed treatment delay status.

Table S11. Summary statistics for 420 patients matched by propensity score for treatment delay analysis.

Figure S12. Sensitivity analysis for propensity score analysis using different cut-offs to define treatment delay.

Table S12. Citations for primary studies / data sets identified for inclusion in meta-analysis

Table S1. Requested variables for IPD analysis.

variable	description
country	Country site for study , or if multisite, country this individual patient was recruited from.
setting1	Setting patient recruited from; from set {outpatient, inpatient, other, NA}
setting2	Setting patient recruited from; from set {OPD, ED, ward, HDUorITU, other, NA}
year	Year patient was recruited
age	Patient age n years at time of recruitment.
sex	{female, male}
HIVstatus	from {1, 0, NA}
CD4	CD4 count cells/mm3
admissionDate	Date of admission to hospital if inpatient
recruitmentDate	Date patient recruited to study
venepunctureDate	Date blood taken for culture (if multiple, date of first)
incubationDate	Date blood received in lab / started culture
positive.cultureDate	Date blood culture flagged positive (MAY INCLUDE CONTAMINANTS / NON-MTB GROWTH)
assayBC	Type of blood culture assay from set {solid, liquid}
volumeBC	Accept measured (actual) or protocol (intended)
numberBC	Number of BCs taken {0,1,2 ….}
failedBC	Number of MTB BC that failed for technical reasons (clotted, not enough volume) {0,1,2 … n} where n = numberBC
contamBC	Number of MTB BC grew a NON-PATHOGEN organism {0,1,2 … m} where m= numberBC- failedBC
other.pathogen.BC	At least one BC grew a non-MTB pathogenic organism; set = organism name; if availableBC == 0 , set to NA {NA, MAC, Spnemon, NTS, MSSA….}
availableBC	Number of MTB BCs for which an uncontaminated result is available = numberBC – failedBC – contamBC {0,1 … }
BCresult	From set {0,1,NA} (availableBC > 0 & at least 1 BC is positive for MTB) set to 1; (availableBC > 0 & no BC positive for MTB) set to 0; (availableBC ==0) set to NA
sputumAvailable	A minimum of 1 sputum processed with available result for either Xpert or liquid culture from {1, 0, NA}. Indeterminate Xpert or contaminated culture = 0. In the case were there are sputum samples from routine care AND from study protocol, this should include only samples obtained for the study.
sputumNumber	Number of sputum collected in study; use measured (actual) or protocol (intended) {0,1,2,3…}
sputumResult	Any positive sputum result for Xpert, or culture if no Xpert done, as defined by WHO criteria (i.e. a positive ID of AFBs as MTB by presence of cording, antigen positivity, molecular typing…) from {1, 0, NA}
sputumXpert	Any positive sputum geneXpert result from {1, 0, NA}
sputumCulture	Any positive sputum culture result (as above) from {1, 0, NA}
ulamAvailable	A minimum of 1 urine processed for LAM from {1, 0, NA}. Interminate ELISA = "0", but <grade 2 on lateral flow = negative LAM (see next)
ulamResult	Any urine LAM at grade 2 or above on lateral flow or equivalent from lab based testing from {1, 0, NA}
cough	Symptom recorded as present, from {1 = present, 0 = absent, NA = not recorded}
fever	Symptom recorded as present, from {1 = present, 0 = absent, NA = not recorded}
weightloss	Symptom recorded as present, from {1 = present, 0 = absent, NA = not recorded}
nightsweats	Symptom recorded as present, from {1 = present, 0 = absent, NA = not recorded}
temperature	Symptom recorded as present, from {1 = present, 0 = absent, NA = not recorded}
RR	Recorded respiratory rate (any or highest)
HR	Recorded heart rate (any or highest)
sBP	Recorded systolic BP (any or lowest)
dBP	Recorded diasystolic BP (any or lowest)

GCS	Recorded Glasgow Coma Scale (any or lowest) {3…15}
AVPU	Recorded AVPU score (any or lowest) {A, V, P, U}
encephalopathy	Is there any acute cognitive or consciousness impairment (by primary study definition)? {1,0} or GCS <15 or AVPU < A?
ambulant	Is the patient able to walk unaided? {1,0} Accept ECOG<3 or GCS>11 or Karnofsky > 40 as proxy.
WHOscreen	Score out of 4 for: cough; fever; weight loss; night sweats {0…4}; if any missing observations, give total out of available (and record number missing in $missingWHOscreen variable)
missingWHOscreen	Number of observations out of the 4 component variables which were NA {0,1,2,3,4}
WHOdanger	Are any of the following present: respiratory rate above 30; temperature above 39.0oC; heart rate above 120 beats per minute; inability to walk unaided {1,0}.
missingWHOdanger	Number of observations out of the 4 component variables which were NA {0,1,2,3,4}
lactate	Venous or arterial accepted, mmol/L
WCC	Peripheral white cell count x109/L
sepsis	Any 2 SIRS criteria: HR > 90 bpm; T > 38C or < 36C, RR > 20, 12<WCC<4
missingSepsis	Number of observations out of the 4 component variables which were NA {0,1,2,3,4}
severe.sepsis	Any 2 SIRS criteria: HR > 90 bpm; T > 37.5C or < 35.5C, RR > 20, 12<WCC<4, plus organ dysfunction()= any one of SBP < 90mmHg, altered mentation(GCS < 15 or AVPU < A, RR > 30] {1,0}
dateDeath	Date of patient death if died during follow-up
inpatientDeath	From {1 = recorded as occurred, 0 = recorded as not occurred , NA = not recorded}
day30death	From {1 = recorded as occurred, 0 = recorded as not occurred , NA = not recorded}
day60death	From {1 = recorded as occurred, 0 = recorded as not occurred , NA = not recorded}
day90death	From {1 = recorded as occurred, 0 = recorded as not occurred , NA = not recorded}
censorDate	Date of last follow up or death
TBdiagnosis	Was there a final diagnosis of TB (by primary study definitions) {1,0}
priorTBRx	Was the patient already on TB therapy >24h prior to blood culture (actual or by protocol)? {1,0}
dateTBRx	Date first dose of any TB Rx
spont.pos	1 = a spontaneous sputum was MTB on Xpert (or if no Xpert done, was MTB on MGIT); 0 = no spontaneous sputum result proving TB (ie no sample, IND Xpert, NEG Xpert, or if no Xpert available, no growth or contam on MGIT)
induc.pos	1 = an induced sputum was MTB on Xpert (or if no Xpert done, was MTB on MGIT); 0 = no induced sputum result proving TB (ie no sample, IND Xpert, NEG Xpert, or if no Xpert available, no growth or contam on MGIT)
ART	On antiretroviral therapy = 1
haemoglobin	In g/dL

Table S2. Bias assessment questionnaire adapted from QUADAS-2 tool.

Domain	Signalling question	Brazil_2004	India_2008	Malawi_2012	Malawi_2013	S.E.Asia_2010	SouthAfrica_2001	SouthAfrica_2006	SouthAfrica_2009	SouthAfrica_2014	SouthAfrica_2015	SouthAfrica_2017	SouthAfrica_2018	Tanzania_2011	Tanzania_2012	Uganda_2009	Uganda_2013	Uganda_2014	Vietnam_2004	Zambia_2014	Zambia_2017
Patient selection	Would every HIV positive patient with at least one WHO TB screening symptom (cough, night sweats, fever, weight loss) in the study setting have an equal chance of recruitment?	*	Y	N	N	Y	N	Y	N	Y	Y	Y	N	N	N	N	N	Y	Y	N	N
	Did the study INCLUDE patients unable to produce sputum?	*	Y	Y	Y	Y	Y	Y	Y	Y	Y	N	Y	Y	Y	Y	Y	Y	Y	Y	Y
	Did the study INCLUDE patients with GCS < 15 (e.g. unresponsive patients unable to give consent at time of recruitment)?	*	N	N	N	N	N	N	N	N	N	Y	Y	N	Y	Y	Y	N	Y	Y	Y
	Was patient selection independent of higher level clinical decision making (e.g. decision to recruit was NOT based on a doctor classifying the patient as having a high probability of TB, or excluding other likely diagnoses, after an overall clinical assessment)?	*	N	Y	Y	Y	N	N	N	Y	Y	Y	N	Y	Y	N	N	Y	Y	N	N
	Did the study NOT exclude patients who were severely unwell (e.g. a very high respiratory rate)?	*	Y	Y	Y	Y	Y	N	Y	N	Y	Y	Y	Y	Y	Y	Y	N	Y	Y	Y
Reference test	Did all the patients receive the same MTB blood culture test?	*	Y	Y	Y	Y	Y	Y	Y	Y	Y	Y	Y	Y	Y	Y	Y	Y	Y	Y	Y
	Did LESS than 10% of recruited patients have no blood culture result available (due to sample loss, culture bottle stock outs, contamination, or other technical failures)?	*	Y	Y	Y	Y	Y	Y	Y	Y	Y	Y	Y	Y	Y	Y	Y	Y	N	Y	Y
	Did the mycobacterial culture facility have quality assurance procedures in place at time of study?	*	Y	Y	Y	Y	Y	Y	Y	Y	Y	Y	Y	Y	Y	Y	Y	Y	Y	Y	Y
Recording of co-factors	Were danger sign variables (respiratory rate, heart rate, temperature, ability to walk unaided) assessed prospectively as part of the study design, as opposed to recorded from routine clinical data / patient notes? (If these variables were not collected in the study please enter "NA")	*	-	-	Y	Y	Y	Y	Y	Y	-	Y	Y	Y	Y	Y	Y	Y	Y	Y	Y
	Were danger sign variables (respiratory rate, heart rate, temperature, ability to walk unaided) available in >90% of recruited patients?	*	N	N	Y	Y	N	N	Y	N	N	Y	Y	Y	Y	Y	Y	N	Y	Y	Y
	Was CD4 count assessed prospectively as part of the study design, as opposed to recorded from routine clinical data / patient notes?	*	N	Y	Y	Y	Y	Y	Y	Y	N	Y	Y	Y	Y	Y	Y	Y	Y	Y	Y
	Did the lab performing CD4 count have quality assurance procedures in place at time of study?	*	Y	Y	Y	Y	Y	Y	Y	Y	Y	Y	Y	Y	Y	Y	Y	Y	Y	Y	Y
Index tests	Was there a dedicated study protocol / staff for collecting sputum samples (rather than relying on standard-of-care / routine care samples)? (If sputum result variables were not collected in the study please enter "NA")	*	-	Y	Y	Y	Y	Y	Y	Y	Y	Y	Y	Y	N	-	-	Y	Y	-	-
	Was sputum induction available?	*	-	Y	N	N	N	Y	N	Y	Y	Y	Y	N	Y	-	-	Y	Y	-	-
	Were collected sputum samples always processed (inoculated for culture or prepared for GneXpert testing) within 24 hours of collection? (If sputum result variables were not collected in the study please enter "NA")	*	-	Y	Y	Y	Y	Y	Y	Y	Y	Y	Y	Y	Y	-	-	Y	Y	-	-
	Did LESS than 20% of sputum samples sent for culture have contamination? (If sputum result variables were not collected in the study please enter "NA")	*	-	Y	Y	Y	Y	Y	Y	Y	Y	Y	Y	Y	Y	-	-	Y	Y	-	-
	Did the lab processing sputums (culture or Xpert) have quality assurance procedures in place at time of study? (If sputum result variables were not collected in the study please enter "NA")	*	-	Y	Y	Y	Y	Y	Y	Y	Y	Y	Y	Y	Y	-	-	Y	Y	-	-

	Were urine LAM tests performed without knowledge of other TB diagnostic tests? (If urine LAM not included in study please enter "NA")	*	-	-	-	-	-	-	Y	Y	Y	Y	Y	-	-	-	-	Y	-	-	-
	Was there any quality assurance of LAM results (e.g. blinded, double reading)? (If urine LAM not included in study please enter "NA")	*	-	-	-	-	-	-	Y	Y	Y	Y	Y	-	-	-	-	Y	-	-	-
**Mortality outcome**	Were there dedicated study procedures for mortality data collection (study staff visit, phone calls) or multiple cross reference of data bases?	*	-	Y	Y	-	Y	Y	Y	Y	Y	Y	Y	Y	Y	Y	Y	Y	N	Y	Y
	Do LESS than 10% of patients have missing outcome data / loss to follow up by day 30 post recruitment?	*	-	Y	N	-	N	N	N	N	Y	Y	Y	N	Y	Y	Y	N	Y	Y	Y

Footnotes:

Y = Yes; N = No; * = unknown / no reply from authors; - = not applicable

Standardised tools are not available for the assessment of quality, bias, and applicability for individual patient data meta analyses. We therefore used a modified QUADAS-2 approach with added domains to assess the risk of bias to our meta-analysis conclusions caused by missing data, and the classification of mortality. Primary study co-authors completed responses to the designed signalling questions, which were then summarised by domain (supplementary figure 1).

Table S3. Measures of model fit and variance / heterogeneity explained in mixed-effect models predicting probability of MTB-BSI

Model Statistic	Description
LRT_{null}	Likelihood-Ratio test p-value testing the hypothesis that the new model has no better fit than the null model.
$LRT_{preceding}$	Likelihood-Ratio test p-value testing the hypothesis that the new model has no better fit than the previous iteration model, i.e. that the added variable has not improved fit more than would be expected by chance alone. If preceding model was constructed with a larger dataset, it was re-fitted with the same reduced dataset as used for the current model so that the models were nested.
Tau squared (τ^2)	Measures variance in the random effects, i.e. it describes variance arising from systematic differences between the primary studies, after adjustment for fixed effect cofactors.[25,26]
Variance Partition Co-efficient (VPC)	Measures proportion of residual individual variation arising from systematic differences between primary studies after adjusting for fixed effect cofactors in the model. The 'latent variable' method was used, which assumes that the binary outcome results from a dichotomised underlying (latent) continuous variable, which follows a logistic probability distribution.[25,27]
$R^2_{marginal}$	Measures proportion of total variance explained by fixed effects. Calculated using *r.squaredGLMM()* function of R package *MuMIn*.[28-30]
$R^2_{conditional}$	Measures variance explained by the complete model – i.e. by fixed and random effects. Calculated using *r.squaredGLMM()* function of R package *MuMIn*.[28-30]
ROC AUC	Area under the receiver operating characteristic curve capturing the within-sample prediction accuracy of the complete model (fixed and random effects). Calculated using the model predicted probabilities compared to the observed outcome.
ΔAUC	Measures the importance of clustering by primary study after adjusting for fixed effect variables. A model containing only fixed-effects variables (no random effect by primary study), and a mixed-effect model containing the same as fixed effects plus random effects by primary study, are made, and the difference in ROC-curve AUC between these two models is calculated.[25]

References

20. Austin PC, Merlo J. Intermediate and advanced topics in multilevel logistic regression analysis. *Stat Med* 2017; **36**(20): 3257-77.

21. Snijders TABB, R.J. Multilevel analysis: An introduction to basic and advanced multilevel modelling. 2nd ed. London: Sage; 2012.

22. Weinmayr G, Dreyhaupt J, Jaensch A, Forastiere F, Strachan DP. Multilevel regression modelling to investigate variation in disease prevalence across locations. *Int J Epidemiol* 2017; **46**(1): 336-47.

23. Barton K. MuMIn: Multi-Model Inference. R package. 1.40.0. ed; 2017.

24. Johnson PC. Extension of Nakagawa & Schielzeth's R(2)GLMM to random slopes models. *Methods Ecol Evol* 2014; **5**(9): 944-6.

25. Nakagawa S, Johnson PCD, Schielzeth H. The coefficient of determination R(2) and intra-class correlation coefficient from generalized linear mixed-effects models revisited and expanded. *J R Soc Interface* 2017; **14**(134).

Figure S1. Summary risk of bias assessment results by included primary dataset and domain.

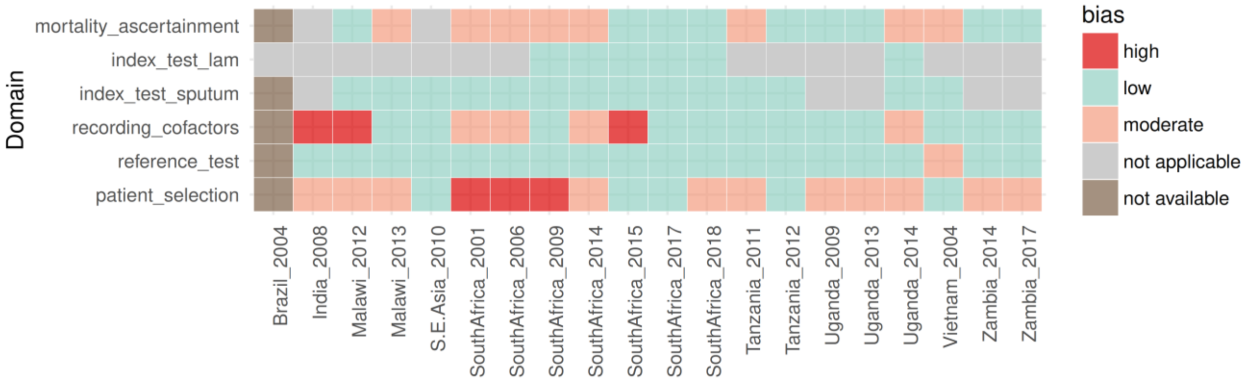

Footnotes:
Signalling question answers shown in supplementary table 1 were summarised by domain as follows:

- Patient selection: number of yes answers > 3 → Low risk of bias
  number of yes answers = 3 → Moderate risk of bias
  number of yes answers < 3 → High risk of bias
- Reference standard: number of yes answers = 3 → Low risk of bias
  number of yes answers = 2 → Moderate risk of bias
  number of yes answers < 2 → High risk of bias
- Recording co-factors: number of yes answers = 4 → Low risk of bias
  number of yes answers = 3 → Moderate risk of bias
  number of yes answers < 3 → High risk of bias
- Index test SPUTUM: number of yes answers > 3 → Low risk of bias
  number of yes answers = 3 → Moderate risk of bias
  number of yes answers < 3 → High risk of bias
- Index test urine-LAM: number of yes answers = 2 → Low risk of bias
  number of yes answers = 1 → Moderate risk of bias
  number of yes answers = 0 → High risk of bias
- Mortality ascertainment: number of yes answers = 2 → Low risk of bias
  number of yes answers = 1 → Moderate risk of bias
  number of yes answers = 0 → High risk of bias

Figure S2. Proportion data missing by variable and primary dataset.

	Brazil 2004	India 2008	Malawi 2012	Malawi 2013	S.E.Asia 2010	SouthAfrica 2001	SouthAfrica 2006	SouthAfrica 2009	SouthAfrica 2014	SouthAfrica 2015	SouthAfrica 2017	SouthAfrica 2018	Tanzania 2011	Tanzania 2012	Uganda 2009	Uganda 2013	Uganda 2014	Vietnam 2004	Zambia 2014	Zambia 2017
Sex	0	0.17	0	0	0	0	0	0	0	0	0	0	0	0	0	0	0	0	0	0
Age	0	0.17	0.01	0	0	0	0	0	0	0	0	0	0	0	0	0	0.04	0	0	0
Danger signs	1	1	1	0	0	0.02	0	0	0	1	0	0	0	0	0	0	0	0	0	0
Sputum result #	1	0.17	0.07	0.12	0.01	0	0.1	0.22	0.01	0.59	0	0.18	0.15	1	1	1	0.04	0.28	1	1
urine-LAM result	1	1	1	1	1	1	1	0	0	0.02	0.33	0.12	1	1	1	1	0	1	0.79	0.73
Final TB diagnosis	0	0	0	0	0	0	0	0	0	0	0	0	0	0	0.87	0.36	0	0	0.62	0.56
Start date TB treatment	1	1	0.75	0.63	1	1	0.01	0.45	0.7	1	0.36	0.11	1	1	1	1	0.72	0	0.71	0.56
TB treatment started prior to blood culture	0	0	0	0	0	0	0	0.45	0	0	0	0	1	0	0.01	0.03	0	0	0.71	0.56
Early death*	1	1	0.06	0.1	1	0	0.13	0.06	0.05	0	0	0.01	0.56	0.01	0	0	0.03	0.18	0	0
Follow-up censor date	1	1	0.06	0.01	1	0.64	0.04	0.02	0	0.01	0	0	0.55	0.01	0	0	0	0	0	0

* Early death defined as death by day 30 or inpatient death if primary study follow-up was less than 30 days.
[#] Sputum result refers to aggregate variable of Xpert result or culture result if Xpert not available.

Table S4. Characteristics of patients meeting IPD inclusion criteria by included primary dataset.

Primary study	n	Female (%)	Median age (IQR)	Median CD4 (IQR)	Inpatient (%)	Danger sign positive (%)	MTB blood culture positive (%)	Final tuberculosis diagnosis$ (%)	Early mortality* (%)
Brazil 2004	44	14 (32%)	34 (31-40)	45 (22-102)	44 (100%)	-	13 (30%)	22 (50%)	-
India 2008	36	3 (10%)	32 (28-38)	230 (194-285)	0 (0%)	-	12 (33%)	18 (50%)	-
Malawi 2012	411	250 (61%)	35 (30-42)	129 (49-221)	0 (0%)	-	11 (3%)	45 (11%)	25 (6%)
Malawi 2013	90	28 (31%)	36 (29-43)	94 (48-232)	90 (100%)	73 (81%)	9 (10%)	38 (42%)	7 (9%)
S.E. Asia 2010	1338	648 (48%)	32 (27-38)	216 (69-373)	40 (3%)	91 (7%)	32 (2%)	335 (25%)	-
South Africa 2001	44	18 (41%)	36 (29-41)	68 (37-134)	44 (100%)	18 (42%)	15 (34%)	26 (59%)	4 (9%)
South Africa 2006	141	92 (65%)	32 (26-38)	107 (38-220)	35 (25%)	24 (17%)	33 (23%)	125 (89%)	9 (7%)
South Africa 2009	264	184 (70%)	35 (29-43)	82 (24-179)	264 (100%)	235 (89%)	34 (13%)	160 (61%)	27 (11%)
South Africa 2014	483	303 (63%)	35 (28-41)	154 (82-242)	203 (42%)	0 (0%)	46 (10%)	201 (42%)	8 (2%)
South Africa 2015	338	201 (59%)	35 (29-42)	132 (51-276)	338 (100%)	-	41 (12%)	123 (36%)	27 (8%)
South Africa 2017	444	293 (66%)	36 (30-42)	88 (35-210)	444 (100%)	444 (100%)	109 (25%)	240 (54%)	36 (8%)
South Africa 2018	615	317 (52%)	36 (31-44)	59 (21-134)	615 (100%)	377 (61%)	209 (34%)	536 (87%)	89 (15%)
Tanzania 2011	230	152 (66%)	36 (30-42)	74 (20-222)	230 (100%)	27 (12%)	40 (17%)	78 (34%)	42 (41%)
Tanzania 2012	145	94 (65%)	39 (32-47)	110 (34-233)	145 (100%)	77 (53%)	12 (8%)	32 (22%)	25 (17%)
Uganda 2009	98	65 (66%)	34 (27-42)	34 (5-98)	98 (100%)	94 (96%)	13 (13%)	13 (100%)	37 (38%)
Uganda 2013	315	167 (53%)	35 (27-40)	49 (13-132)	315 (100%)	304 (97%)	76 (24%)	203 (100%)	102 (32%)
Uganda 2014	479	305 (64%)	32 (28-39)	97 (22-288)	338 (71%)	0 (0%)	53 (11%)	199 (42%)	18 (4%)
Vietnam 2004	61	10 (16%)	30 (25-40)	20 (9-98)	61 (100%)	31 (51%)	8 (13%)	30 (49%)	19 (38%)
Zambia 2014	58	33 (57%)	34 (28-40)	49 (24-107)	58 (100%)	54 (93%)	27 (47%)	21 (95%)	36 (62%)
Zambia 2017	117	53 (45%)	34 (29-42)	60 (21-141)	117 (100%)	100 (85%)	35 (30%)	52 (100%)	62 (53%)
Total, all datasets	5751	3230 (56%)	34 (28-41)	109 (34-249)	3479 (60%)	1949 (40%)	828 (14%)	2497 (46%)	573 (14%)

Footnotes:

Denominator for percentages is from number meeting IPD inclusion criteria minus number with missing observations on the variable.

* Early mortality defined as death by day 30 or inpatient death if primary study follow-up was less than 30 days, in all patients meeting IPD inclusion criteria irrespective of final tuberculosis diagnosis.$ Final tuberculosis diagnosis variable defined as per respective primary study case definitions.

Table S5. Nested mixed-effect models examining predictors of tuberculosis blood culture result and heterogeneity between datasets.

Iteration	Random effects	Fixed effects	number datasets	n	LRT_{null} p-value	$LRT_{preceding}$ p-value*	τ^2	VPC	$R^2_{marginal}$	$R^2_{conditional}$	ROC AUC	Δ AUC
0	Intercept by primary data set	None (null model)	20	5751	NA	NA	0·79	0·19	0	0·19	0·75	0·25
1	Intercept by primary data set	CD4 count	20	5751	<0.0001	<0.0001	0·82	0·2	0·13	0·30	0·81	0·07
2	Intercept by primary data set	CD4 count + presence of danger signs	16	4921	<0.0001	<0.0001	0·60	0·15	0·18	0·31	0·81	0·04
3	Intercept by primary data set	CD4 count + presence of danger signs + hospitalisation	16	4921	<0.0001	<0.0001	0·56	0·14	0·28	0·39	0·82	0·03
4	Intercept by primary data set	CD4 count + presence of danger signs + hospitalisation + TB treatment prior to blood culture	15	4454	<0.0001	<0.0001	0·58	0·14	0·30	0·40	0·83	0·03
5	Intercept by primary data set	CD4 count + presence of danger signs + hospitalisation + TB treatment prior to blood culture + number of blood cultures performed	15	4454	<0.0001	0.00019	0·59	0·14	0·30	0·41	0·83	0·02
6	Intercept by primary data set	CD4 count + presence of danger signs + hospitalisation + TB treatment prior to blood culture + number of blood cultures performed + final diagnosis was TB	15	4224	<0.0001	<0.0001	0·49	0·13	0·69	0·73	0·91	0·01

Footnotes:

Model summary measures defined in table 1. Models fitted to raw (unimputed) data.

Adding ART status or year of recruitment to study to the final model does not improve model fit ($LRT_{proceeding}$ p-value 0.442 and 0.271 respectively), between study heterogeneity (τ^2 0.71 and 0.46; VPC 0.18 and 0.13 respectively), variance explained ($R^2_{marginal}$ 0.67 and 0.68; $R^2_{conditional}$ 0.59 and 0.58 respectively), or within-sample discriminatory predictive accuracy (ROC AUC 0.91 and 0.91; ΔAUC 0.00 and 0.00 respectively).

* When model had less included cases than the preceding model (due to missing observations in added co-variate), the preceding model was re-fitted using complete cases for the current model to allow Likelihood Ratio testing of nested model

Table S6. Availability of TB diagnostic tests stratified by study.

Study	n	Positive TB blood culture (%)	Sputum TB culture available (%)	Sputum TB culture positive (%)	Sputum Xpert available (%)	Sputum Xpert positive (%)	Urine LAM available (%)	Urine LAM positive (%)
Brazil_2004	44	30%	0%	ND	0%	ND	0%	ND
India_2008	36	33%	83%	33%	0%	ND	0%	ND
Malawi_2012	411	3%	93%	11%	0%	ND	0%	ND
Malawi_2013	90	10%	88%	46%	78%	41%	0%	ND
S.E.Asia_2010	1338	2%	99%	17%	0%	ND	0%	ND
SouthAfrica_2001	44	34%	93%	68%	0%	ND	0%	ND
SouthAfrica_2006	141	23%	90%	64%	0%	ND	0%	ND
SouthAfrica_2009	264	13%	78%	34%	0%	ND	100%	35%
SouthAfrica_2014	483	10%	99%	36%	0%	ND	100%	11%
SouthAfrica_2015	338	12%	41%	25%	41%	26%	98%	16%
SouthAfrica_2017	444	25%	92%	51%	100%	48%	67%	40%
SouthAfrica_2018	615	34%	82%	59%	82%	62%	85%	40%
Tanzania_2011	230	17%	86%	32%	0%	ND	0%	ND
Tanzania_2012	145	8%	0%	ND	0%	ND	0%	ND
Uganda_2009	98	13%	0%	ND	0%	ND	0%	ND
Uganda_2013	315	24%	0%	ND	0%	ND	0%	ND
Uganda_2014	479	11%	96%	35%	0%	ND	100%	14%
Vietnam_2004	61	13%	72%	50%	0%	ND	0%	ND
Zambia_2014	58	47%	0%	ND	0%	ND	21%	50%
Zambia_2017	117	30%	0%	ND	0%	ND	27%	28%
**TOTAL**	**5751**	**14%**	**77%**	**33%**	**20%**	**51%**	**42%**	**25%**

Footnotes:

ND = not done.

Figure S3. Distribution of positive, negative and unavailable sputum TB diagnostic testing (A) and urine-lipoarabinomannan testing (B) stratified by study in patients with MTB-BSI.

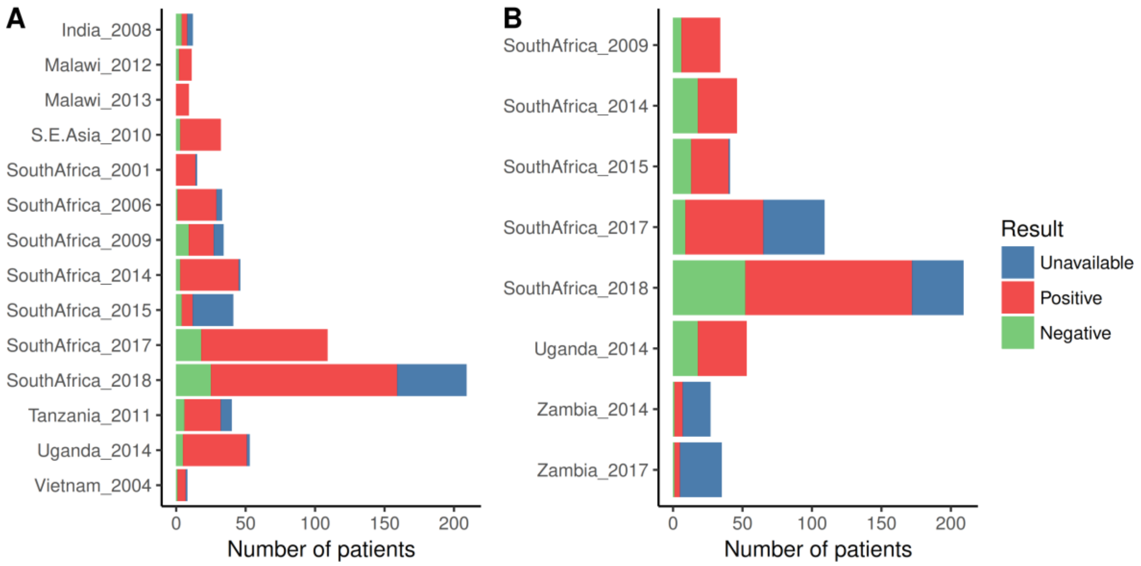

Footnotes:

Included are patients with positive TB blood culture from studies which also collected IPD sputum (**A**) or urine-LAM (**B**) TB diagnostic tests. Sputum culture was used as a surrogate for Xpert in studies which did not perform Xpert testing of sputum (10 of 14 studies). Shown are number of MTB-BSI patients who had positive, negative, or unavailable sputum or urine-LAM test, giving an indication of how many of these patients could have been diagnosed by rapid sputum or urine testing. Diagnostic yield is seen to vary substantially between studies largely due to marked variation in proportion with unavailable test. Plots are generated from raw (unimputed) data.

Figure S4. Diagnostic yield of rapid diagnostics in patients with MTB-BSI.

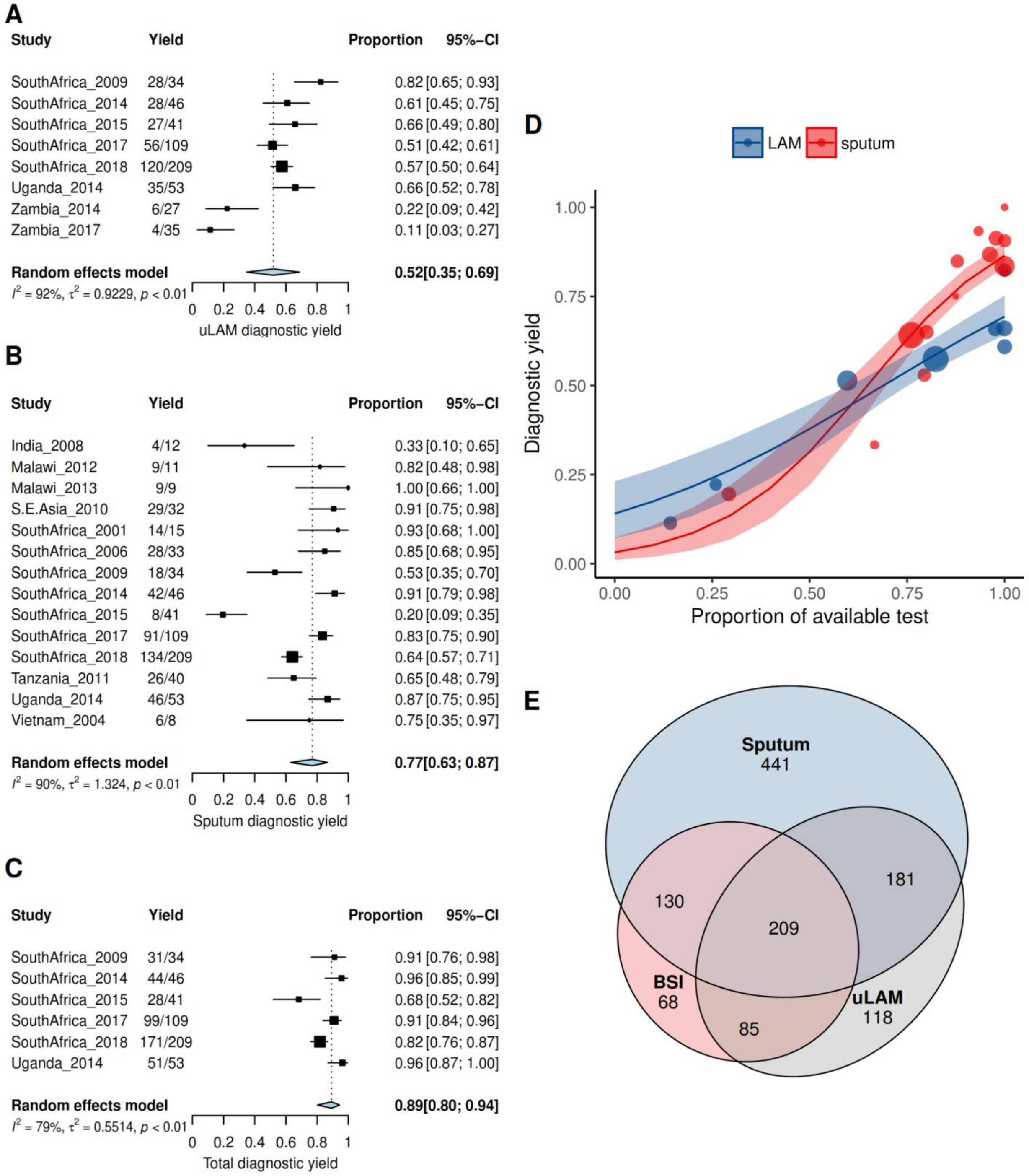

Footnotes:

Pooled diagnostic yield of urine-LAM (A), sputum (B) and both (C) for MTB-BSI; meta regression on availability of test (D) showing diagnostic yield of studies (points, where size of point is proportional to study size) as a function of proportion of available test for LAM (blue) and sputum (red) with lines showing model population estimates and shaded areas 95% confidence intervals; Venn diagram of number of positive tests in studies performing both urine-LAM testing and sputum testing (E). In all cases sputum variable was Xpert (or culture result as surrogate if Xpert not available). Analyses use raw (unimputed) data.

Figure S5. Sensitivity analysis exploring effect of composite sputum variable on diagnostic yield analyses.

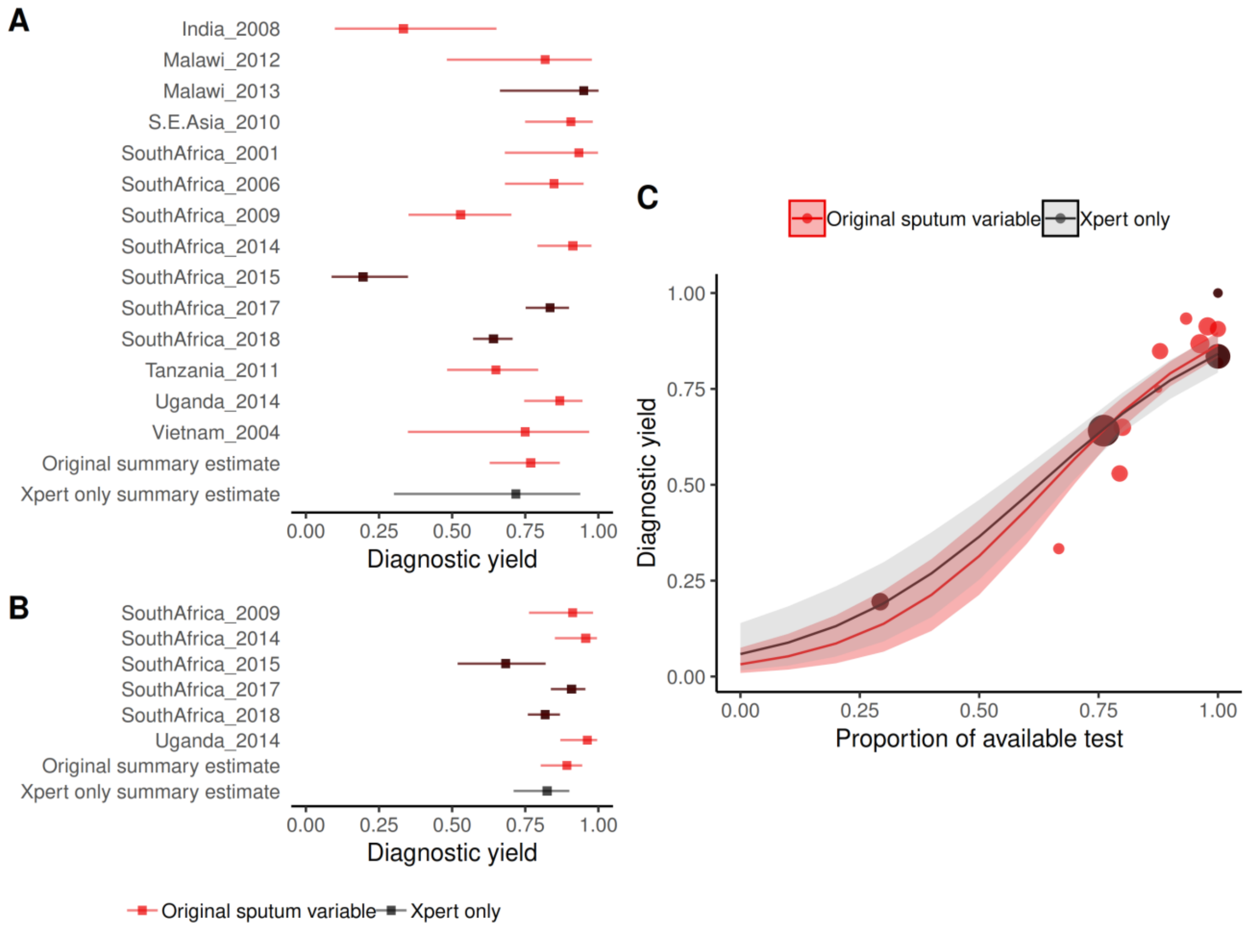

Footnotes:

All analyses described in main text were repeated but replacing the composite sputum variable with only those studies where Xpert results were available. In all cases, the red represents the original analysis and the grey, the Xpert-only analysis. A: Pooled diagnostic yield of sputum Xpert for MTB BSI (4 studies, 1487 participants) 72% (95% CI 30- 94%). B: Pooled composite diagnostic yield of sputum Xpert and uLAM for MTB BSI (3 studies, 1397 participants) 82% (95% CI 71% - 90%). In both A and B, point estimates are squared, and 95% confidence intervals whiskers; C: Meta-regression of diagnostic yield as a function of proportion of available Xpert test result. Actual diagnostic yield from individual studies plotted as points (with size proportional to number of participants included in analysis) and model estimates of population diagnostic yield plotted as lines, with 95% confidence intervals indicated by shaded areas. In all cases summary estimates from the sensitivity analysis fall within the confidence intervals of the primary analysis. Analyses use raw (unimputed) data.

Table S7: Associations of no sputum result available

	N obs	Unadjusted					N datasets	Adjusted for clustering by dataset				
		OR	95%CI			pvalue		OR	95%CI			pvalue
Walks unaided	1859	0.41	0.29	to	0.57	0.00000	9	0.52	0.35	to	0.77	0.00100
Encephalopathic	977	2.63	1.67	to	4.09	0.00002	5	4.17	2.53	to	6.88	0.00000
Sepsis	1483	1.03	0.73	to	1.45	0.87107	10	0.91	0.62	to	1.35	0.65118
Severe sepsis	1350	1.23	0.84	to	1.78	0.27693	7	1.68	1.12	to	2.53	0.01266
Danger signs	1945	1.96	1.43	to	2.68	0.00003	11	1.62	1.11	to	2.37	0.01212
MTB BSI	2131	1.82	1.39	to	2.38	0.00001	14	1.69	1.25	to	2.31	0.00080
Early mortality	1687	2.74	1.90	to	3.91	0.00000	12	2.71	1.78	to	4.11	0.00000
log CD4 count	2131	0.87	0.79	to	0.96	0.00663	14	0.93	0.83	to	1.04	0.20357
Age, per 10 years	2124	1.04	0.90	to	1.20	0.57781	14	1.04	0.88	to	1.22	0.65606
Tachypnoeic	1742	0.95	0.57	to	1.51	0.83815	9	1.44	0.82	to	2.53	0.20939
Pulse, per 10bpm	1318	1.01	0.94	to	1.09	0.75512	6	1.03	0.95	to	1.12	0.44950
Hypotensive	1017	0.76	0.50	to	1.14	0.19013	6	0.70	0.46	to	1.06	0.09467
log blood lactate	528	2.11	1.34	to	3.35	0.00133	1	-		-		-

Table S8: Associations of no urine-LAM result available

	N obs	Unadjusted					n datasets	Adjusted for clustering by dataset				
		OR	95%CI			p-value		OR	95%CI			p-value
Walks unaided	1334	0.20	0.14	to	0.29	<0.00001	7	0.39	0.25	to	0.60	0.00002
Encephalopathic	949	4.17	2.79	to	6.22	<0.00001	5	2.63	1.63	to	4.25	0.00007
Sepsis	950	1.63	1.05	to	2.63	0.03708	5	0.89	0.53	to	1.49	0.65336
Severe sepsis	950	3.51	2.44	to	5.10	<0.00001	5	1.87	1.15	to	3.03	0.01195
Danger signs	1350	5.64	3.73	to	8.80	<0.00001	7	2.38	1.43	to	3.96	0.00087
MTB BSI	1473	1.76	1.24	to	2.47	0.00135	8	2.18	1.44	to	3.31	0.00026
Early mortality	1449	5.22	3.54	to	7.66	<0.00001	8	2.18	1.33	to	3.58	0.00203
log CD4 count	1473	0.86	0.76	to	0.98	0.02158	8	0.88	0.75	to	1.03	0.11321
Age, per 10 years	1468	1.15	0.96	to	1.37	0.12704	8	1.09	0.88	to	1.33	0.43182
Tachypnoeic	1346	3.67	2.53	to	5.31	<0.00001	7	1.47	0.85	to	2.53	0.16406
Pulse, per 10bpm	947	1.16	1.08	to	1.26	0.00014	5	1.14	1.03	to	1.27	0.01157
Hypotensive	945	1.94	1.35	to	2.78	0.00029	5	0.72	0.44	to	1.18	0.19769
log blood lactate	576	3.60	2.44	to	5.40	<0.00001	2	1.93	1.20	to	3.09	0.00627

Notes:
Encephalopathic = GCS<15 or AVPU < 4; sepsis & severe sepsis by Sepsis-2 definitions; early mortality = death in hospital or by 30-days follow-up; tachypnoeic = respiratory rate > 30 per minute; hypotensive = systolic BP < 100 mmHg.
Unadjusted estimates from univariable logistic regression; adjusted estimates are fixed-effects from mixed-effects logistic regression including random-intercept by primary dataset. OR = odds ratio of no available index test (sputum or urine); 95%CI estimated from fixed-effect standard errors (* +/- 1.96). All analyses use raw (unimputed) data.

Table S9. Characteristics of patients with a final TB diagnosis in each primary study included in meta-analysis.

Study	n	MTB BSI (%)	Inpatient (%)	Age/years (median [IQR])	Male (%)	CD4 cells/mm^3 (median [IQR])	ART (%)	WHO Danger Signs (%)	Sputum Positive (%)	uLAM Positive	Early death
Brazil_2004	22	59%	100%	32 (30-37)	68%	42 (17-75)	36%	NR	ND	ND	NR
India_2008	18	67%	0%	30 (27-36)	94%	216 (187-255)	NR	NR	56%	ND	NR
Malawi_2012	45	24%	0%	35 (30-40)	51%	93 (49-216)	0%	NR	96%	ND	20%
Malawi_2013	38	24%	100%	34 (30-41)	74%	74 (44-135)	NR	79%	79%	ND	11%
S.E.Asia_2010	335	9%	6%	32 (28-38)	61%	101 (30-251)	4%	18%	50%	ND	NR
SouthAfrica_2001	26	54%	100%	38 (29-41)	54%	62 (48-126)	0%	50%	92%	ND	12%
SouthAfrica_2006	125	26%	26%	32 (26-39)	36%	118 (42-248)	1%	18%	65%	ND	NR
SouthAfrica_2009	160	21%	100%	34 (29-42)	29%	74 (20-153)	6%	89%	43%	51%	4%
SouthAfrica_2014	201	23%	54%	35 (29-42)	43%	145 (61-255)	NR	0%	85%	27%	NR
SouthAfrica_2015	123	33%	100%	33 (27-39)	36%	70 (30-172)	37%	NR	28%	41%	10%
SouthAfrica_2017	240	44%	100%	35 (29-41)	34%	74 (29-162)	31%	100%	85%	40%	9%
SouthAfrica_2018	536	39%	100%	36 (31-44)	49%	57 (21-122)	NR	62%	58%	36%	NR
Tanzania_2011	78	51%	100%	36 (31-43)	44%	44 (7-146)	22%	9%	82%	ND	48%
Tanzania_2012	32	38%	100%	39 (31-44)	44%	38 (16-114)	34%	62%	ND	ND	23%
Uganda_2009	13	100%	100%	33 (32-39)	31%	8 (3-47)	100%	100%	ND	ND	54%
Uganda_2013	203	37%	100%	35 (28-41)	46%	45 (12-119)	84%	98%	ND	ND	33%
Uganda_2014	199	27%	76%	32 (28-39)	42%	61 (16-185)	NR	NR	80%	35%	NR
Vietnam_2004	30	27%	100%	30 (23-38)	83%	20 (7-78)	3%	53%	70%	ND	53%
Zambia_2014	21	29%	100%	35 (25-40)	38%	40 (28-98)	NR	95%	ND	5%	71%
Zambia_2017	52	13%	100%	34 (29-43)	52%	72 (22-208)	NR	87%	ND	8%	50%
**TOTAL**	**2497**	**31%**	**76%**	**34 (29-41)**	**46%**	**71 (24-172)**	**NR**	**51%**	**56%**	**22%**	**21%**

Footnotes:

ART = antiretroviral therapy (at baseline); MTB BSI = MTB bloodstream infection; uLAM = urinary LAM

TB diagnosis was defined as per respective primary study definitions rather than being recoded with a harmonised case definition.

Figure S6: Time varying coefficient of presence of MTB -BSI fitted with natural spline with 4 degrees of freedom

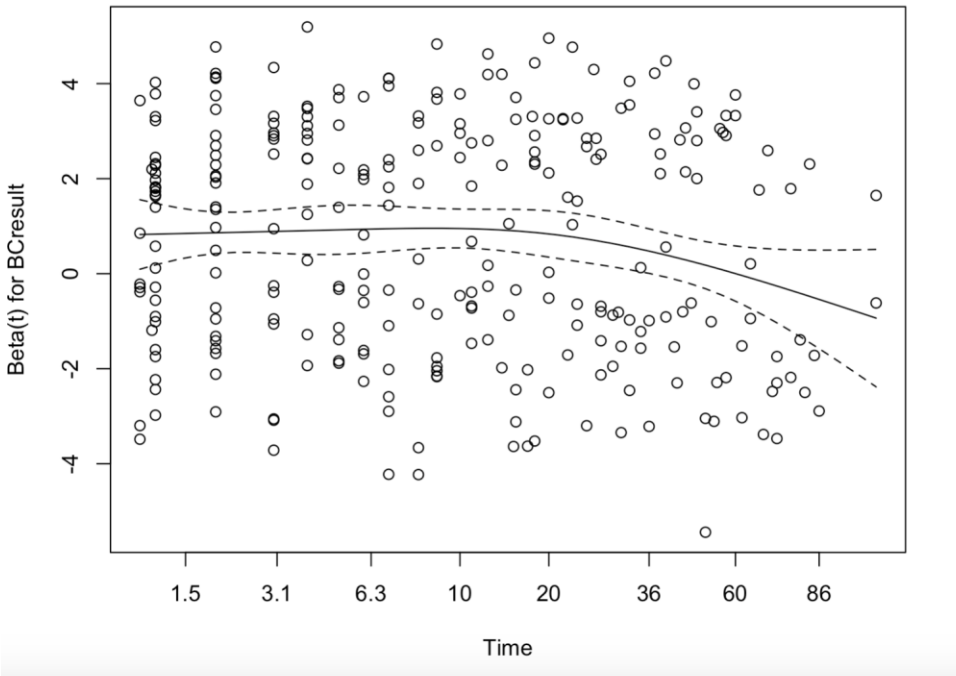

Figure S7. Mortality-hazard associated with positive TB blood culture and urine-LAM in studies performing both tests.

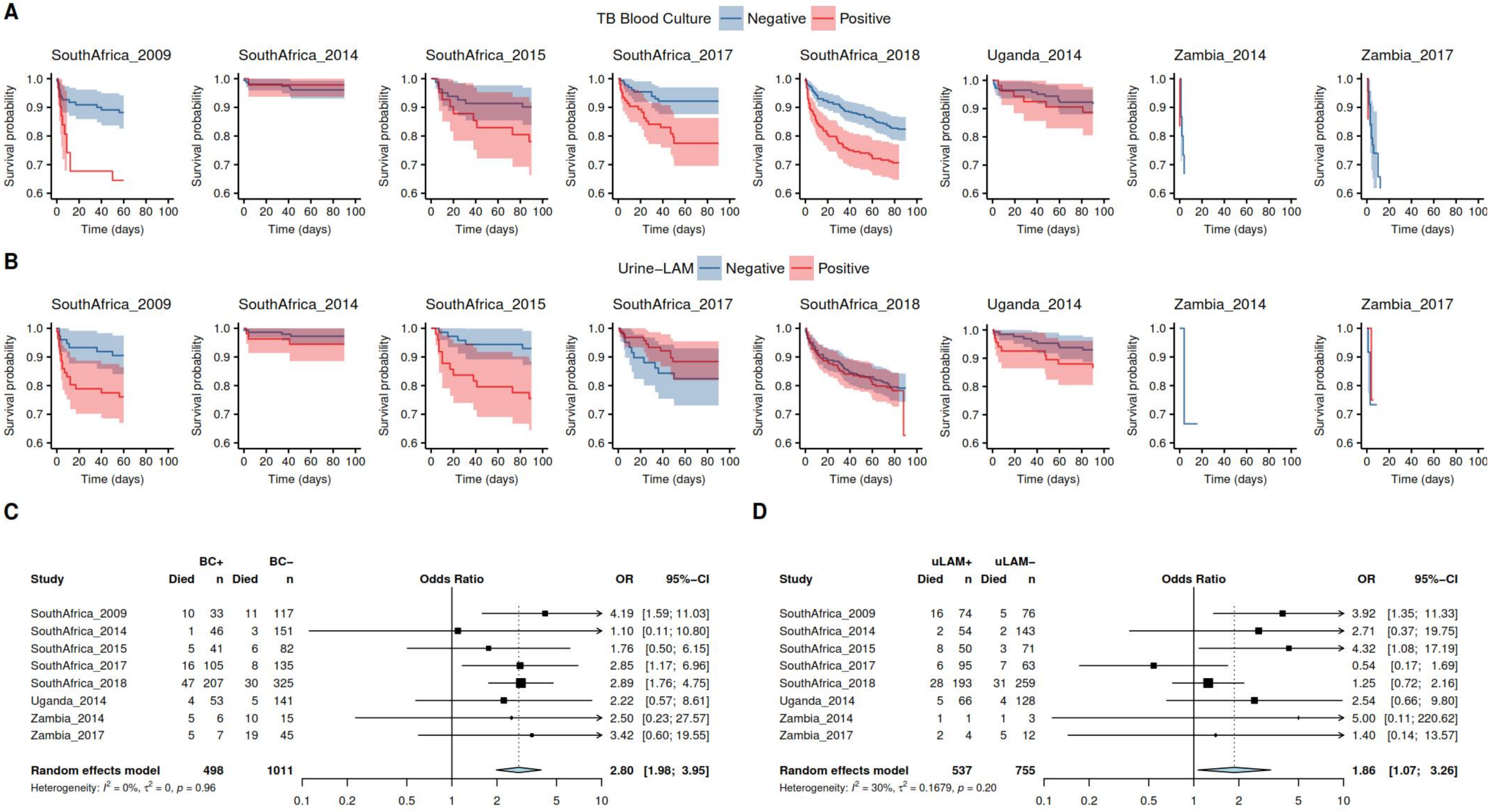

Footnotes:

Eight studies performed urine-LAM in addition to TB blood culture. Complete-case (not imputed) Kaplan Meir plots showing survival by test result are shown for TB blood culture (**A**) and urine-LAM (**B**). Unadjusted HRs for mortality are shown for TB blood culture positive (**C**) and urine-LAM positive (**D**) patients by primary study, and pooled by two-stage meta-analysis random-effects model. Analyses are generated from raw (unimputed) data.

Table S10. Adjusted hazard ratio of death in urine-LAM positive patients with diagnosis of TB.

Covariate	Summary HR	95% CI
Urine-LAM result	1.24	0.86 – 2.36
Age (per 5 years increase)	1.13	1.03 – 1.18
One or more WHO danger signs	1.90	0.91 – 13.68
CD4 count (per 100 cell/ microliter increase)	0.78	0.54 – 1.01
ART at baseline	1.26	0.76 – 1.88
Male sex (vs female) before 30 days	1.45	1.08 – 2.43
Male sex (vs female) after 30 days	0.69	0.41 – 0.93

Footnotes:

This model was a post-hoc analysis, in which urine-LAM status was substituted for TB blood culture status as a mortality predictor (cf. table 5 in main manuscript). Summary hazard ratios from Cox proportional hazard model using *a priori* covariates; setting (inpatient vs outpatient) excluded because the dataset includes almost exclusively inpatients. Missing data imputed using mixed effect models (5 datasets) and 95% confidence intervals constructed from quantiles of 1000 pooled replicates from each imputed dataset.

Figure S8. Risk of death by treatment delay and patient group: raw data and propensity score analysis.

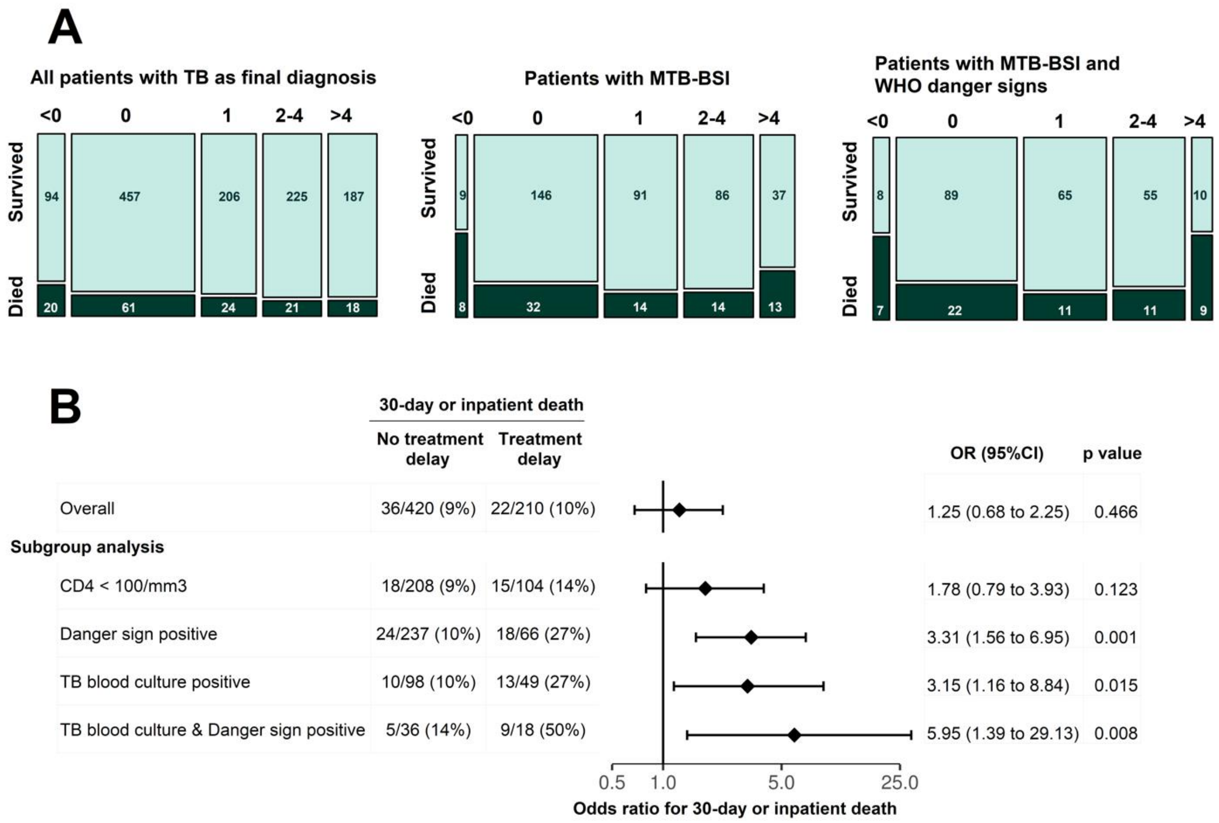

Footnotes:

A. Plots are proportional representations of contingency tables made from all IPD aggregated across primary datasets with available data by complete-case analysis. This shows raw data for survival to discharge from hospital or 30-days follow-up (survived versus died) by time-to-ATT category (days between blood culture collection and ATT start: <0 i.e. before enrolment, 1, 2-4, or >4 days). This is shown for 3 patient groups: the whole cohort of patients who had a final tuberculosis diagnosis (left panel), the subgroup of patients with a positive tuberculosis blood culture (middle panel), and the subgroup who had both a positive tuberculosis blood culture and ≥1 WHO danger sign (right panel). The higher mortality risk seen in patients with no delay in ATT (<0 or 0 days) was hypothesised to represent more urgent initiation of therapy in patients perceived to be more critically-ill, a confounder of the relationship between time-to-antimicrobial and risk of mortality (full assumed causal structure shown in a Directed Acyclic Graph, Figure S9).

B. To adjust for this hypothesised confounding a propensity-score analysis was performed with patients matched by propensity for delayed start of ATT, here defined as >4 days between blood culture collection and ATT start. In this matched cohort, odds ratios for death associated with treatment delay were greater than 1 in more unwell subgroups, specifically in patients with MTB-BSI. Other cut-offs for defining treatment delay were explored in a sensitivity analysis (Figure S12 in supplementary appendix).

Figure S9. Directed Acyclic Graph (DAG) explicating assumptions made about causal structure for propensity score analysis of effect treatment delay on mortality.

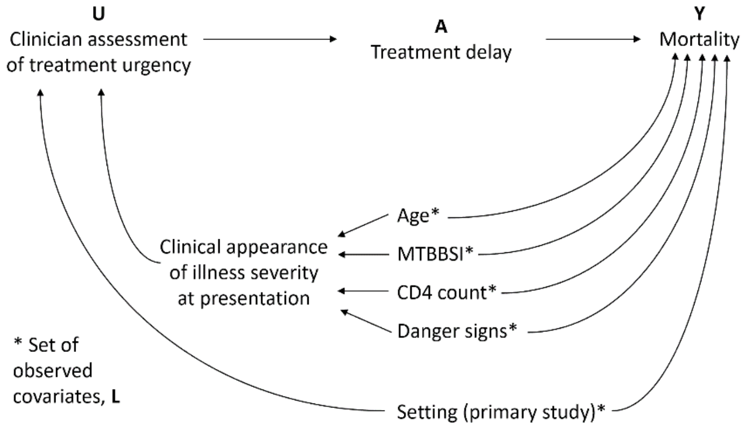

Footnotes

We hypothesised that a causal relationship between treatment delay and mortality in HIV-associated tuberculosis (A → Y) is biased by the confounding represented by a backdoor path mediated through the unobserved variable "clinician assessment of treatment urgency" (U). We further hypothesised that U was likely, in turn, caused by an observed variable set, L (Age, MTBBSI, CD4 count, presence of danger signs, and primary study setting). The implication of these assumptions is that matching patients based on a propensity score for A given L can eliminate the confounding mediated by U, giving a less biased estimate of A → Y. Finally, we hypothesised that the effect A → Y would be more pronounced in the presence of more severe disease, defined by subgroups with CD4<100, presence of ≥1 danger signs, & MTBBSI (this hypothesised interaction effect is not shown on DAG).

Figure S10. Patient inclusion in treatment delay analysis.

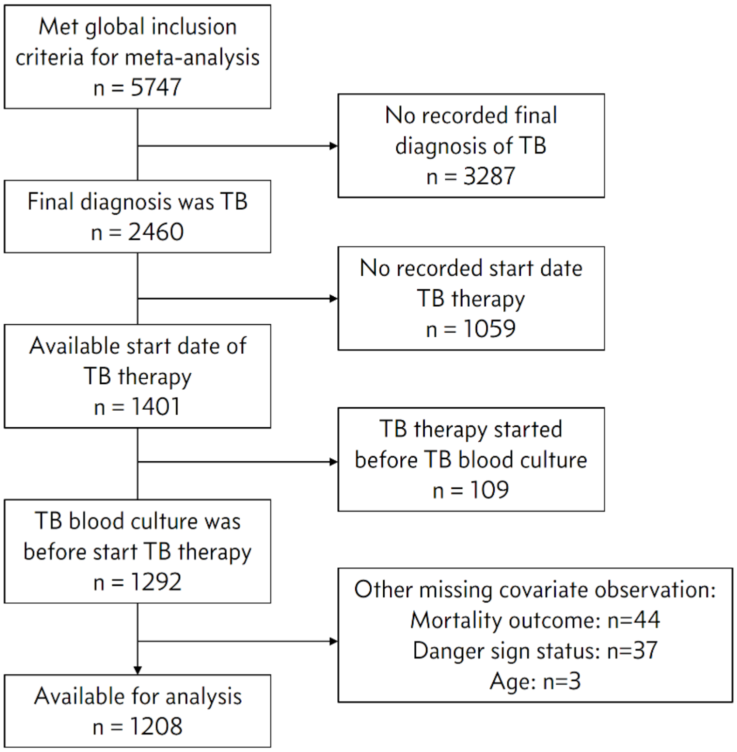

Footnotes:

1208 patients met inclusion criteria, of whom 630 could be matched on propensity score for treatment delay in a 2:1 ratio (420 patients without treatment delay : 210 with treatment delay).

Figure S11. Distribution of propensity score by observed treatment delay status.

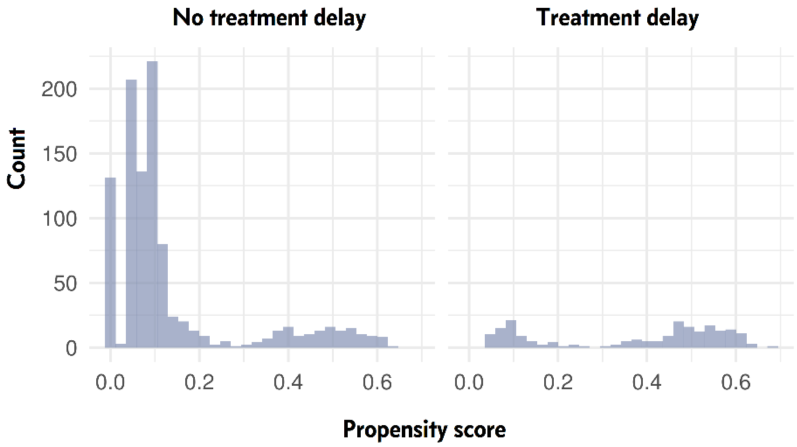

Table S11. Summary statistics for 630 patients matched by propensity score for treatment delay analysis.

	No treatment delay (n=420)	Treatment delay (n=210)	Total (N=630)	p value
**Age (scaled)**				< 0.001
median	0.222	-0.205	0.111	
IQR	-0.519, 0.969	-0.843, 0.530	-0.632, 0.852	
**logCD4_scaled**				0.972
median	0.356	0.360	0.356	
IQR	-0.325, 0.936	-0.480, 0.911	-0.359, 0.935	
**WHO danger signs**				< 0.001
0	183 (43.6%)	144 (68.6%)	327 (51.9%)	
≥1	237 (56.4%)	66 (31.4%)	303 (48.1%)	
**TB blood culture**				1.000
Negative	322 (76.7%)	161 (76.7%)	483 (76.7%)	
Positive	98 (23.3%)	49 (23.3%)	147 (23.3%)	

Figure S12. Sensitivity analysis for propensity score analysis using different cut-offs to define treatment delay.

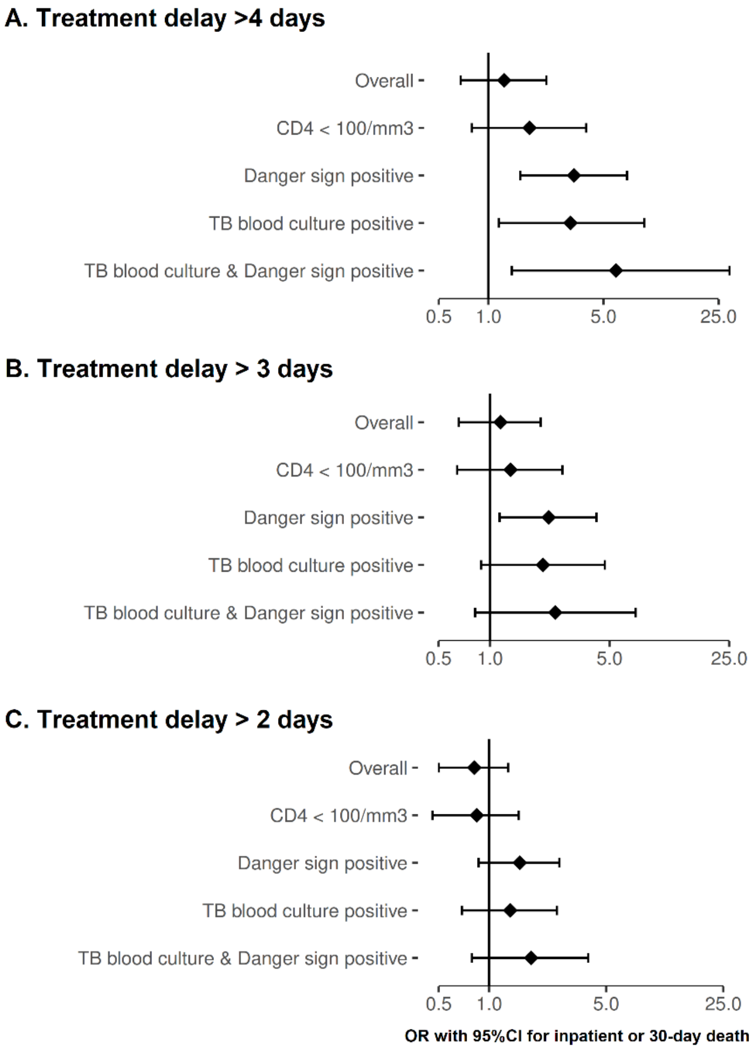

Footnotes:
In main manuscript treatment delay was defined as >4 days from blood culture collection to start of anti-tuberculosis therapy, and associated risk of mortality estimated using propensity score matched cohort analysis, including subgroups of interest (TB blood culture positive, WHO danger sign positive). This analysis is reproduced here (**A**), and the analysis is repeated with two different cut-off values for defining treatment delay: >3 days (**B**) and >2 days (**C**). The effect size is seen to be sensitive to the cut-off used, with longer delay associated with larger effect size i.e. greater increased risk of mortality from longer delay. All analyses used complete case analysis.

Table S12. Citations for primary studies / data sets identified for inclusion in meta-analysis

Brazil 2004	Bacha HA, Cimerman S, de Souza SA, Hadad DJ, Mendes CM. Prevalence of mycobacteremia in patients with AIDS and persistant fever. *Braz J Infect Dis* 2004; **8**(4): 290-5.
Brazil 1997	Grinsztejn B, Fandinho FC, Veloso VG, et al. Mycobacteremia in patients with the acquired immunodeficiency syndrome. *Arch Intern Med* 1997; **157**(20): 2359-63.
S.E.Asia 2010	Varma JK, McCarthy KD, Tasaneeyapan T, et al. Bloodstream infections among HIV-infected outpatients, Southeast Asia. *Emerg Infect Dis* 2010; **16**(10): 1569-75.
India 2008	Gopinath K, Kumar S, Singh S. Prevalence of mycobacteremia in Indian HIV-infected patients detected by the MB/BacT automated culture system. *Eur J Clin Microbiol Infect Dis* 2008; **27**(6): 423-31.
Ivory Coast 1993	Vugia DJ, Kiehlbauch JA, Yeboue K, et al. Pathogens and predictors of fatal septicemia associated with human immunodeficiency virus infection in Ivory Coast, west Africa. *J Infect Dis* 1993; **168**(3): 564-70.
Kenya 1995	Gilks CF, Brindle RJ, Mwachari C, et al. Disseminated Mycobacterium avium infection among HIV-infected patients in Kenya. *J Acquir Immune Defic Syndr Hum Retrovirol* 1995; **8**(2): 195-8.
Malawi 2012	Bedell RA, Anderson ST, van Lettow M, et al. High prevalence of tuberculosis and serious bloodstream infections in ambulatory individuals presenting for antiretroviral therapy in Malawi. *PLoS One* 2012; **7**(6): e39347.
Malawi 2013	Feasey NA, Banada PP, Howson W, et al. Evaluation of Xpert MTB/RIF for detection of tuberculosis from blood samples of HIV-infected adults confirms Mycobacterium tuberculosis bacteremia as an indicator of poor prognosis. *J Clin Microbiol* 2013; **51**(7): 2311-6.
South Africa 2015	Lawn SD, Kerkhoff AD, Burton R, et al. Rapid microbiological screening for tuberculosis in HIV-positive patients on the first day of acute hospital admission by systematic testing of urine samples using Xpert MTB/RIF: a prospective cohort in South Africa. *BMC Med* 2015; **13**: 192.
South Africa 2009	Shah M, Variava E, Holmes CB, et al. Diagnostic accuracy of a urine lipoarabinomannan test for tuberculosis in hospitalized patients in a High HIV prevalence setting. *J Acquir Immune Defic Syndr* 2009; **52**(2): 145-51.
South Africa 2001	von Gottberg A, Sacks L, Machala S, Blumberg L. Utility of blood cultures and incidence of mycobacteremia in patients with suspected tuberculosis in a South African infectious disease referral hospital. *Int J Tuberc Lung Dis* 2001; **5**(1): 80-6.
South Africa 2018	Schutz C, Barr D, Andrade BB, et al. Clinical, microbiologic, and immunologic determinants of mortality in hospitalized patients with HIV-associated tuberculosis: A prospective cohort study. PLoS Med. 2019;16(7):e1002840.
South Africa 2017	Griesel R, Stewart A, van der Plas H, et al. Optimizing Tuberculosis Diagnosis in Human Immunodeficiency Virus-Infected Inpatients Meeting the Criteria of Seriously Ill in the World Health Organization Algorithm. Clin Infect Dis. 2018;66(9):1419-1426.
South Africa 2006	Wilson D, Nachega J, Morroni C, Chaisson R, Maartens G. Diagnosing smear-negative tuberculosis using case definitions and treatment response in HIV-infected adults. *Int J Tuberc Lung Dis* 2006; **10**(1): 31-8.
South Africa 2014	Nakiyingi L, Moodley VM, Manabe YC, et al. Diagnostic accuracy of a rapid urine lipoarabinomannan test for tuberculosis in HIV-infected adults. *J Acquir Immune Defic Syndr* 2014; **66**(3): 270-9.

Uganda 2014	Nakiyingi L, Moodley VM, Manabe YC, et al. Diagnostic accuracy of a rapid urine lipoarabinomannan test for tuberculosis in HIV-infected adults. *J Acquir Immune Defic Syndr* 2014; **66**(3): 270-9.
Tanzania 2012	Crump JA, Ramadhani HO, Morrissey AB, et al. Bacteremic disseminated tuberculosis in sub-saharan Africa: a prospective cohort study. *Clin Infect Dis* 2012; **55**(2): 242-50.
Tanzania 2011	Munseri PJ, Talbot EA, Bakari M, Matee M, Teixeira JP, von Reyn CF. The bacteraemia of disseminated tuberculosis among HIV-infected patients with prolonged fever in Tanzania. *Scand J Infect Dis* 2011; **43**(9): 696-701.
Vietnam 2004	Louie JK, Chi NH, Thao le TT, et al. Opportunistic infections in hospitalized HIV-infected adults in Ho Chi Minh City, Vietnam: a cross-sectional study. *Int J STD AIDS* 2004; **15**(11): 758-61.
Uganda 2009	Jacob ST, Moore CC, Banura P, et al. Severe sepsis in two Ugandan hospitals: a prospective observational study of management and outcomes in a predominantly HIV-1 infected population. *PLoS One* 2009; **4**(11): e7782.
Uganda 2013	Jacob ST, Pavlinac PB, Nakiyingi L, et al. Mycobacterium tuberculosis bacteremia in a cohort of hiv-infected patients hospitalized with severe sepsis in uganda-high frequency, low clinical suspicion [corrected] and derivation of a clinical prediction score. *PLoS One* 2013; **8**(8): e70305.
Zambia 2014	Andrews B, Muchemwa L, Kelly P, Lakhi S, Heimburger DC, Bernard GR. Simplified severe sepsis protocol: a randomized controlled trial of modified early goal-directed therapy in Zambia. *Crit Care Med* 2014; **42**(11): 2315-24.
Zambia 2017	Andrews B, Semler MW, Muchemwa L, et al. Effect of an Early Resuscitation Protocol on In-hospital Mortality Among Adults With Sepsis and Hypotension: A Randomized Clinical Trial. *JAMA* 2017; **318**(13): 1233-40.
